# Efficacy and Safety of Carpal Tunnel Release in Patients Aged 70 Years and Older: A Systematic Review and Meta-Analysis

**DOI:** 10.3390/medsci14020264

**Published:** 2026-05-20

**Authors:** Elisa Di Dio, Giulia Maria Sassara, Adriano Cannella, Federico Ianniccari, Gabriele Delia, Vitale Cilli, Marco Valerio, Giulia Frittella, Lorenzo Rocchi, Rocco De Vitis

**Affiliations:** 1 Hand Surgery Unit, Department of Orthopedics, Fondazione Policlinico Universitario Agostino Gemelli IRCCS, 00168 Roma, Italy; elisa.didio01@icatt.com (E.D.D.); giuliamariasassara@gmail.com (G.M.S.); adriano.cansa@gmail.com (A.C.); marco.valerio01@icatt.it (M.V.);; 2 Department of Orthopaedics and Traumatology, CTO Hospital, 10126 Turin, Italy; federico.ianniccari@edu.unito.it; 3 Department of Plastic and Reconstructive Surgery, University Hospital of Messina “AOU Gaetano Martino”, 98124 Messina, Italy; gablieledelia@gmail.com; 4 Department of Chirurgie de la Main, Centre Hospitalier Interregional Edith Cavell, 1160 Bruxelles, Belgium

**Keywords:** carpal tunnel syndrome/surgery, decompression, surgical, aged, treatment outcome, patient satisfaction, peripheral nerve injuries

## Abstract

**Background:** Carpal tunnel syndrome (CTS) is the most prevalent peripheral nerve entrapment neuropathy, with rising incidence in aging populations. Uncertainty persists regarding the efficacy and safety of carpal tunnel release (CTR) in patients aged ≥ 70 years. **Objectives:** To systematically evaluate the indications, clinical outcomes, and utility of CTR in elderly patients (≥70 years), with comparison to younger cohorts. **Methods**: Following PRISMA 2020 guidelines, PubMed/MEDLINE, Scopus, CENTRAL, Embase, Web of Science, and grey literature sources were searched from inception through September 2025. Two independent reviewers extracted data; inter-rater agreement was strong (κ = 0.81–0.86). The primary outcome was the Boston Carpal Tunnel Questionnaire (BCTQ). Weighted mean differences (WMDs) with 95% confidence intervals (CIs) were calculated using DerSimonian–Laird random-effects models. Certainty of evidence was assessed using the GRADE framework. **Results:** A total of 20 studies encompassing 3841 operated hands, including 1139 hands in elderly patients and 2702 hands in younger comparators across comparative studies, were analyzed. Mean SS-BCTQ improvement was 1.8 points (95%CI: 1.6–2.0; exceeding the established MCID of 1.04–1.05 points). FS-BCTQ improvement was 1.1 points (95%CI: 0.9–1.3; marginally below the pooled MCID of 1.13 points). Elderly patients demonstrated SS-BCTQ improvement of 1.7 points and satisfaction rates of 72–94%, comparable to younger cohorts (75–95%; *p* = 0.38). Grip strength improved 15–25% in younger patients but remained unchanged in elderly patients (*p* < 0.001). Sensory recovery reached 42% in elderly versus 58% in younger patients (*p* < 0.01). Complication rates were low and age-independent (3.1%; RR 1.08; 95%CI: 0.86–1.35; *p* = 0.52). GRADE certainty was as follows: low for symptom and functional improvement; very low for surgery versus conservative management. **Conclusions:** CTR is associated with significant symptomatic benefit in elderly patients when conservative treatment fails, with complication rates comparable to younger populations. Age alone should not constitute a surgical contraindication. Preoperative counseling must establish realistic expectations regarding grip strength and functional recovery. High-quality randomized trials in elderly populations remain an urgent research priority.

## 1. Introduction

Carpal tunnel syndrome is the most common peripheral nerve entrapment neuropathy, affecting 1–5% of the general population [1]. The condition exhibits a bimodal age-specific incidence, with an initial peak between 45 and 60 years (approximately 275 per 100,000 annually) and a second peak between 70 and 85 years, at which epidemiological data suggest annual incidence rates in the range of 400–650 per 100,000, derived across UK surveillance and review data [2,3]. The lifetime incidence of carpal tunnel surgery is substantial and continues to rise in parallel with demographic aging trends [4]. Established risk factors include female sex, obesity, diabetes mellitus, hypothyroidism, and repetitive occupational hand use [5,6]. According to the United Nations World Population Prospects 2024, the global population aged 65 and older, currently estimated at approximately 830 million, is projected to reach approximately 1.7 billion by 2054 [4], rendering this a progressively significant clinical and public health challenge as the elderly population more than doubles over the coming three decades.

Surgical decompression via CTR has been established as a standard treatment for CTS across all age groups, with reported success rates of 70–95% [7,8]. Diagnosis relies on validated clinical criteria [9] corroborated by electrodiagnostic testing, with evidence-based management algorithms provided by the American Academy of Orthopaedic Surgeons (AAOS) 2025 Clinical Practice Guideline [10], developed for adult patients aged ≥ 18 years generally, without specific recommendations targeted to the ≥70 age subgroup, which superseded the previous 2016 edition and incorporates updated evidence on both operative and non-operative management, including recommendations with strong evidence that mini-open and endoscopic CTR provide equivalent long-term patient-reported outcomes [10]. Despite this consolidated evidence base, important uncertainty persists regarding the optimal management of elderly patients compared with younger cohorts. Elderly individuals characteristically present with more advanced disease, with the mean duration of symptoms before surgical consultation ranging from 18 to 46 months in elderly cohorts versus 6 to 12 months in younger patients [11,12,13]. This diagnostic delay is largely attributable to the misattribution of hand symptoms to normal aging processes by both patients and clinicians [14,15,16,17,18].

The high prevalence of medical comorbidities in elderly populations further complicates surgical decision-making. Diabetes mellitus affects 25–36% of elderly CTS patients compared with 8–12% in younger groups [12,19], osteoarthritis is present in 43–46% versus 8–15% [19,20], and cardiovascular and hypertensive disease occurs substantially more frequently in elderly cohorts [12,21]. The impact of diabetes on CTR outcomes has been specifically investigated, with evidence suggesting that diabetic comorbidity does not preclude favorable postoperative recovery [22,23]. The emergence of SARS-CoV-2 infection as a potential trigger or exacerbating factor for peripheral nerve entrapment syndromes, including CTS, is an additional consideration in the workup of elderly patients presenting with new or worsening symptoms [24]. Furthermore, elderly patients may simultaneously harbor concurrent hand and wrist pathologies, including advanced arthritic conditions such as wrist arthritis amenable to specialist hand surgical intervention, that may necessitate combined or staged procedures requiring careful integrated surgical planning in a dedicated hand surgery unit [25].

The existing literature on CTR outcomes in elderly patients presents contradictory conclusions. Several investigations report results comparable to those of younger cohorts [21,26,27,28], whereas others describe attenuated improvements in both subjective and objective functional measures [7,29]. This discordance likely reflects heterogeneity in outcome instruments and their sensitivity to clinical change [30], variable follow-up duration (6 months to 10 years), differing patient selection criteria, and inconsistent definitions of “elderly” across investigations (ranging from ≥60 to ≥80 years). It should also be noted that a minority of CTS cases with normal nerve conduction studies represent a distinct diagnostic subset that may influence population-level outcome data [31]. Patient-reported outcomes may not align with objective functional assessments, particularly in elderly patients whose functional demands and expectations differ substantially from those of younger individuals [32]. The absence of comprehensive systematic analyses addressing this specific population limits the formulation of evidence-based clinical recommendations.

This systematic review and meta-analysis therefore aimed to synthesize the available evidence regarding the indications for and utility of CTR in elderly patients (defined as ≥70 years), with subgroup comparison to younger cohorts. Four clinical questions were addressed: (1) What clinical and electrodiagnostic characteristics define CTS presentation across age strata? (2) What postoperative outcomes can be expected in both the general CTR population and elderly subgroups specifically? (3) How do outcomes compare between elderly and younger patients? (4) What factors should inform surgical decision-making and patient counseling in elderly patients with CTS?

## 2. Materials and Methods

### 2.1. Literature Search Strategy

A comprehensive systematic literature search was performed in adherence with PRISMA guidelines, interrogating PubMed/MEDLINE and Scopus from inception through September 2025. In compliance with PRISMA 2020 Item 7, which requires full search strategies to be reported for all databases and registers searched, the following additional sources were interrogated using equivalent search strategies adapted to each database’s indexing vocabulary: (1) Cochrane Central Register of Controlled Trials (CENTRAL) via the Cochrane Library; (2) Embase (Elsevier); (3) Web of Science Core Collection (Clarivate Analytics); (4) ClinicalTrials.gov and the WHO International Clinical Trials Registry Platform (ICTRP) for ongoing and unpublished studies; and (5) grey literature sources, including relevant conference proceedings of the American Society for Surgery of the Hand (ASSH) and the British Society for Surgery of the Hand (BSSH).

The search strategy employed MeSH terms and free-text keywords capturing both general CTS populations and elderly-specific cohorts:

(“carpal tunnel syndrome” [MeSH Terms] OR “carpal tunnel syndrome” [Title/Abstract] OR “CTS” [Title/Abstract] OR “median nerve compression” [Title/Abstract] OR “median neuropathy” [Title/Abstract]) AND (“carpal tunnel release” [MeSH Terms] OR “carpal tunnel decompression” [Title/Abstract] OR “CTR” [Title/Abstract] OR “surgical release” [Title/Abstract] OR “endoscopic release” [Title/Abstract] OR “open release” [Title/Abstract] OR “carpal tunnel surgery” [Title/Abstract]) AND (“aged” [MeSH Terms] OR “elderly” [Title/Abstract] OR “older adult” [Title/Abstract] OR “older patient*” [Title/Abstract] OR “geriatric” [Title/Abstract] OR “senior” [Title/Abstract] OR “age” [Title/Abstract] OR “age groups” [Title/Abstract]).

No language restriction was applied to the search strategy. Non-English publications meeting all eligibility criteria were included.

**Figure 1:**
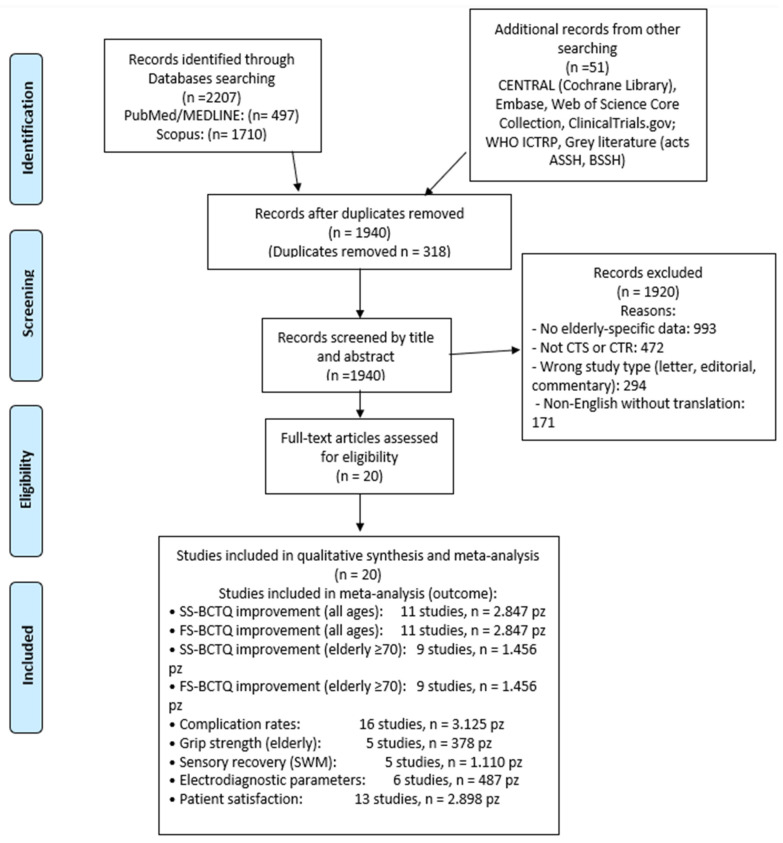
PRISMA 2020 flow diagram illustrating the study selection process.

This search identified 497 articles in PubMed and 1710 articles in Scopus. Searches in CENTRAL, Embase, Web of Science, and grey literature sources identified an additional 51 records. After removal of 318 duplicates and systematic two-stage screening (title/abstract followed by full-text review) conducted by two independent reviewers in adherence with PRISMA 2020 Item 8, 20 studies met eligibility requirements. It must be explicitly noted that this systematic review was not prospectively registered on PROSPERO, INPLASY, or any equivalent registry prior to study selection or data extraction. The absence of prospective registration prevents external verification of pre-specified outcomes, inclusion criteria, and statistical analysis plans; constitutes a formal downgrading consideration in all GRADE certainty assessments (see Table 1 and Section 2.6); and is acknowledged as a significant methodological limitation. All future systematic reviews by this group will be prospectively registered prior to data extraction. The updated PRISMA 2020 flow diagram is presented in Figure 1.

### 2.2. Inclusion and Exclusion Criteria

Studies were eligible for inclusion if they (1) examined patients undergoing CTR with specific reporting of outcomes for elderly patients (≥70 years) either as the primary study population or as a distinct subgroup; (2) reported clinical, functional, or electrodiagnostic outcomes using validated or standardized instruments [9,33]; (3) included a minimum follow-up of six months; and (4) were published in peer-reviewed journals with sufficient methodological detail to permit quality assessment. One post hoc justified protocol deviation, declared transparently, was made: Englert and Hammert [29] were included despite reporting 30-day outcomes, given the unique and otherwise unrepresented PROMIS-domain data provided. Findings from this study are presented exclusively as early postoperative supplementary evidence and are not incorporated into the primary outcome analyses. A second pre-specified protocol deviation is declared: Naves and Kouyoumdjian [34] was included despite enrolling patients who did not undergo CTR (prospective normative electrophysiological study). This study was retained exclusively to provide age-specific normative nerve conduction reference values for elderly patients, which are essential for contextualizing the electrodiagnostic severity data reported in Section 3.2.3. Data from Naves and Kouyoumdjian [34] were not incorporated into any surgical outcome meta-analysis and are presented exclusively as normative reference evidence.

Studies were excluded if they (1) included only patients younger than 70 years without age-stratified analysis; (2) focused exclusively on surgical technique without reporting outcome data; (3) reported follow-up shorter than six months (excluding the post hoc justified protocol deviation, declared transparently); or (4) were case reports, narrative reviews, or editorials without original primary data. As a formal clarification required by peer review: Porter et al. [28] was excluded on criterion (1), as it did not report outcomes for a ≥70-year subgroup as a discrete, data-extractable population group. Data from Porter et al. [28] cited in Section 3.6.2 represent contextual supporting evidence from a non-included study and are explicitly identified as such; this data was not incorporated into any meta-analytic pooling.

The authors acknowledge that, in the absence of a prospectively registered protocol, these deviations cannot be formally verified as pre-specified. They are declared as transparent post hoc decisions, each with explicit methodological justification, and are treated as formal risk-of-bias elements in the GRADE certainty assessments.

### 2.3. Data Extraction and Quality Assessment

Two independent reviewers extracted data using a standardized form capturing both overall and age-stratified subgroup information. Extracted variables included the following: study design; Newcastle–Ottawa Scale (NOS) quality score; patient demographics and symptom duration; clinical and electrodiagnostic disease severity; primary and secondary outcomes for total and elderly subgroups; and complication and revision surgery rates. Inter-rater agreement was assessed using Cohen’s kappa (κ). For title/abstract screening, κ = 0.81 (95%CI: 0.77–0.85), indicating strong agreement. For full-text review, κ = 0.86 (95%CI: 0.79–0.93). Data extraction agreement was 93.4%; all discrepancies were resolved by consensus discussion with a third independent reviewer. A methodological limitation of quality assessment must be acknowledged: the NOS is validated for cohort and case–control studies and is not appropriate for cross-sectional designs. For the three cross-sectional studies included in this review (Blumenthal et al. [12], Study 8; Naves and Kouyoumdjian [34], Study 11; and Aghda et al. [11], Study 16), the validated instrument is the JBI Critical Appraisal Checklist for Analytical Cross-Sectional Studies [35]. NOS scores assigned to these studies for consistency purposes should therefore be interpreted with caution. This limitation was formally considered in GRADE certainty assessments: for outcomes predominantly derived from cross-sectional data, no upgrading was applied.

### 2.4. Outcome Measures

The primary outcome was patient-reported symptom severity and functional status measured using the BCTQ [33], comprising an 11-item SS subscale (SS-BCTQ; score range 1–5) and an 8-item FS subscale (FS-BCTQ; score range 1–5), with lower scores indicating better outcomes. The SS-MCID was originally established at approximately 1.04 points by Ozyurekoglu et al. [36] using ROC curve analysis, with the authors recommending 1.0 as a practical threshold; a pooled estimate of 1.05 points was subsequently confirmed in the systematic review by Mehta et al. [37]. It must be explicitly acknowledged that De Kleermaeker et al. [38] did not report or endorse a fixed threshold of 1.05 points; rather, this study concluded that no single fixed MCID applies universally to all patients, and recommended a proportional approach whereby MCID is calculated individually relative to baseline severity (MCID_SS = 0.46 × baseline SS score; MCID_FS = 0.28 × baseline FS score). Applying this formula to a typical baseline SS score of 3.0–3.2 (as reported in the majority of included studies) yields an MCID_SS of approximately 1.38–1.47 points, which is notably higher than the fixed pooled estimate of 1.05 attributed to Ozyurekoglu et al. [38] and Mehta et al. [37]. All MCID-based interpretations in this review must be read in light of this methodological discrepancy, and the 1.05 threshold should not be exclusively attributed to De Kleermaeker et al. [36]. For the FS-BCTQ, a pooled MCID of 1.13 points has been reported [36,37]; however, it must be acknowledged that no definitive consensus has been established for the FS subscale MCID, and the threshold varies depending on baseline severity, patient population characteristics, and the methodological approach used for its calculation [36,37]. Specifically, individual studies report FS-MCID values ranging from 0.27 to 1.13 points depending on these factors. It should further be noted that applying a uniform MCID threshold derived from general CTS populations to an elderly-specific subgroup with systematically higher baseline scores may underestimate the clinical meaningfulness of observed improvements in this age group, as patients with higher baseline symptom burden may require greater absolute change to perceive clinically important improvement [36,37]. All MCID-based interpretations in this review should be read in light of this uncertainty. The responsiveness of the BCTQ and QuickDASH to clinically meaningful change after CTR has been independently validated [30]. Secondary outcomes included: grip and pinch strength; Semmes–Weinstein monofilament (SWM) sensory testing and two-point discrimination; electrodiagnostic parameters including sensory and motor latencies, conduction velocities, and response amplitudes [34,39]; patient satisfaction (standardized as the proportion reporting satisfied or very satisfied); and complication and revision surgery rates. Validated functional tests beyond standard dynamometry were considered supplementary measures of recovery [40]. The Michigan Hand Outcomes Questionnaire (MHQ) [21] and QuickDASH [41] were analyzed as supplementary patient-reported instruments where available. PROMIS domain scores were analyzed as distinct early postoperative outcome data [29].

### 2.5. Statistical Analysis

Meta-analysis was performed using DerSimonian–Laird random-effects models for outcomes reported across multiple studies with comparable measurement methods. WMDs with 95% CIs were calculated for continuous outcomes. Heterogeneity was assessed using the I^2^ statistic (values > 50% indicating substantial heterogeneity warranting cautious interpretation of pooled estimates). For outcomes with I^2^ > 50%, specifically grip strength (I^2^ = 78%) and sensory response amplitudes (I^2^ = 67%), pooled quantitative estimates are presented as exploratory approximations only and are not appropriate for direct clinical application. Narrative synthesis is provided as the primary summary of evidence for these outcomes. Meta-regression analyses were performed to explore potential sources of heterogeneity, including age threshold definition (≥60, ≥65, ≥70, ≥80 years); surgical technique (open versus endoscopic); and follow-up duration. Subgroup analyses compared elderly (≥70 years) and younger patients for primary and secondary outcomes. Sensitivity analyses were performed to assess the robustness of pooled estimates: (1) excluding the single low-quality study (NOS < 5); (2) restricted to high-quality studies (NOS ≥ 7); (3) excluding the pre-specified methodological exception [29]; and (4) restricted to studies using a uniform elderly threshold of ≥70 years. Publication bias was evaluated using funnel plot inspection and Egger’s test where ≥10 studies were available for a given outcome reported separately for SS-BCTQ and FS-BCTQ. Statistical significance was set at *p* < 0.05. Primary meta-analyses (DerSimonian–Laird random-effects models for SS-BCTQ, FS-BCTQ, complication rates, and patient satisfaction) and forest plot generation were performed using Review Manager 5.4 (The Cochrane Collaboration, London, UK). Forest plots for the primary outcomes, SS-BCTQ improvement in the overall population (Supplementary Figure S1) and in the elderly subgroup ≥ 70 years (Supplementary Figure S2), FS-BCTQ improvement in the overall population (Supplementary Figure S3) and in the elderly subgroup ≥ 70 years (Supplementary Figure S4), and overall complication rates (Supplementary Figure S5), are provided as Supplementary Material. Heterogeneity assessment (I^2^ statistic), meta-regression analyses, Egger’s test for publication bias, and sensitivity analyses were performed using Stata 17.0 (StataCorp LLC, College Station, TX, USA). The unit of analysis throughout is ‘hands’ rather than ‘patients’, consistent with the carpal tunnel release literature. No included study provided explicit data on the number of patients undergoing bilateral simultaneous procedures; consequently, potential within-patient correlation arising from bilateral CTR could not be statistically adjusted for in the pooled analyses. This represents an additional methodological limitation with a specific directional implication: violation of the independence assumption between observations from the same patient results in artificially narrow confidence intervals (i.e., underestimated standard errors), such that the 95% CIs reported for all pooled outcomes in this review may be optimistically narrow. Readers and guideline developers should interpret pooled estimates with awareness that true confidence intervals may be wider than reported.

### 2.6. Assessment of Evidence Certainty

The certainty of evidence for each primary and key secondary outcome was assessed using the GRADE (Grading of Recommendations Assessment, Development and Evaluation) framework [42].

As in the GRADE approach, randomized controlled trials (RCTs) start as high-quality evidence and observational studies as low-quality evidence supporting estimates of intervention effects. Since the entire evidence base of this review comprises observational studies, all outcomes are assigned an initial GRADE certainty of LOW. Certainty was upgraded by one level when (1) a large and consistent magnitude of effect was demonstrated (defined as WMD ≥ 2 × the established MCID threshold); and/or (2) a dose–response gradient was identified. Five factors may lead to rating down the certainty of evidence [42]: (1) serious risk of bias; (2) substantial inconsistency (I^2^ > 50%); (3) indirectness of evidence; (4) imprecision; and (5) probable publication bias.

A GRADE Summary of Findings (SoF) table for the primary outcomes is presented as Table 1.

**Table 1:** GRADE Summary of Findings (SoF). This review was not prospectively registered (PROSPERO/INPLASY/OSF). The absence of pre-registration was considered an additional risk-of-bias element in all GRADE certainty assessments and contributes a formal rationale against upgrading any outcome from its observed level. GRADE ratings may therefore be subject to downward revision pending independent replication in prospectively registered analyses.

Outcome	Number of Studies	Study Design	Anticipated GRADE Certainty	Primary Rationale
SS-BCTQ improvement (all ages)	11	Observational	LOW	Not upgraded. The pre-specified upgrade criterion of WMD ≥ 2 × MCID (i.e., ≥2.10 points [38]) is not formally met (observed WMD = 1.8). I^2^ = 52% constitutes a factor against upgrading per GRADE inconsistency domain, irrespective of partial meta-regression explanation. The observed WMD of 1.8 exceeds the SS-MCID [38] and is clinically large, but these alone are insufficient for upgrading under strict GRADE standards.
FS-BCTQ improvement (all ages)	11	Observational	LOW	Not upgraded: pooled WMD 1.1 marginally does not reach the pooled FS-MCID of 1.13 [36,37] (difference: −0.03 points); upgrade criterion not met. The clinical significance of this marginal shortfall should be interpreted in light of the variability in FS-MCID estimates across populations [36,37].
Complication rates	15	Observational	LOW	No upgrade factor; precise estimates
Grip strength recovery (elderly)	5	Observational	VERY LOW	Downgraded ×2: I^2^ = 78%; 95%CI crosses zero
Sensory recovery (elderly)	5	Observational	LOW	Consistent direction but incomplete
Surgery vs. conservative (elderly)	1	Observational	VERY LOW	Single non-randomized study; risk of confounding by indication

## 3. Results

### 3.1. Study Characteristics and Patient Demographics

The 20 included studies (Table 2) collectively encompassed 3841 operated hands across surgical studies, of which 1139 hands belonged to elderly patients and 2702 hands to younger comparator groups across the 9 comparative studies. It must be noted that no included study constitutes a true randomized controlled trial. Ettema et al. [43] is a population-based comparative effectiveness study in which patients self-selected or were clinically directed to treatment groups and were not randomly allocated. All evidence in this review is therefore derived from observational research. Study 12 (Faour Martín et al. [44], published in Spanish) was extracted by a bilingual reviewer.

Quality assessment using the modified NOS revealed that 14 studies (74%) achieved scores ≥ 7 (high quality), 4 studies (21%) scored 5–6 (moderate quality), and 1 study (5%) scored < 5 (low quality).

The age threshold defining “elderly” varied substantially across investigations, ranging from ≥60 years [28] to ≥80 years [40,45,46,47], with the majority employing ≥65 or ≥70 years as the operative cutoff [11,12,19,20,21,26,27]. The primary meta-analytic estimates for the “elderly” subgroup reported in the main text are those derived from studies employing ≥70 years as the operative cutoff; studies using other thresholds are incorporated in the overall analyses and sensitivity analyses only.

**Table 2:** Included studies. Notes: post hoc justified protocol deviation, declared transparently to ≥6-month follow-up criterion (30-day PROMIS data retained for supplementary analysis only; see Section 2.2). ^‡^ Lowest satisfaction rate in the series, reported in 24 patients aged ≥80 years at 11-month follow-up. ^†^ NOS scores assigned to Studies 8 (Blumenthal), 11 (Naves), and 16 (Aghda) are methodologically limited as NOS is not validated for cross-sectional designs. ^§^ The Levine questionnaire is the original designation of the BCTQ; both names refer to the same validated patient-reported outcome instrument [33]. NR: Not Reported.

No.	First Author	Year	Ref.	NOS Score	Study Design	Hands (*n*)	“Elderly” Definition	Mean Age, Years (Range)	Follow-Up Duration	CTR Type	Outcome Instruments	SS-BCTQ Pre → Post	FS-BCTQ Pre → Post	Grip Strength	EMG/Sensory Testing	Satisfaction Rate
1	Tomaino & Weiser	2001	[26]	5	Retrospective cohort; elderly only	13	≥70 years	76 (70–84)	24 months	Open	BCTQ; subjective global assessment	→≈1.0 (near-normal)	NR	NR	NR	85% (11/13)
2	Leit	2004	[27]	6	Prospective longitudinal; elderly only	14	>70 years	79 (72–90)	12 months	Open	BCTQ; grip strength dynamometry	2.9 → 1.4	1.8 → 0.9	No significant improvement	NR	93%
3	Mondelli	2004	[19]	7	Prospective cohort; elderly + younger comparison	72 elderly; total sample including younger comparators	70–90 years	75.6 (70–90)	6 months	Open	BCTQ; EMG/NCS; sensory testing	3.05 → 1.47	2.80 → 1.67	Less than younger patients	Improved but incomplete normalization	NR
4	Hobby	2005	[7]	8	Prospective cohort; age-stratified	97 total (14 >70 years)	>70 years	53 overall (21–85)	6 months	Open	BCTQ; PEM	>70 years: satisfaction 79% vs. 87% younger	Worse function (numbness, dexterity)	NR	NR	79% (>70 years)
5	Townshend	2005	[13]	7	Retrospective cohort; elderly only	83	≥70 years	78.5 (70–90)	12–49 months (mean 29)	Open	BCTQ; EMG/NCS	Median 1.3 (IQR: 1.1–1.7)	NR	NR	87% showed EMG improvement; 49% achieved normal studies	94%
6	Weber & Rude	2005	[21]	8	Prospective cohort; elderly only	92	≥65 years	74 ± 6 (65–95)	6 months	Open	BCTQ; MHQ; grip/pinch strength; 2-PD	3.05 → 1.60	2.42 → 1.67	No significant change (21.2 → 22.1 kgf; *p* = 0.24)	Improved	83%
7	Wilgis	2006	[48]	9	Prospective multicenter; four age strata	635 total (26 >80 years)	≤40/41–60/61–80/>80 years	84 (>80 years stratum)	6 months	Open	BCTQ; Semmes-Weinstein; grip/pinch strength	>80 years: 3.05 → 1.64 (comparable across age groups)	>80 years: 2.88 → 1.99 (comparable across age groups)	Age-graded improvement: 22%/18%/12%/3%	Improved in all groups; less recovery in >80 years	NR
8	Blumenthal	2006	[12]	8 ^†^ (6/8 JBI score)	Prospective cross-sectional; age-stratified	343 total (70 ≥65 years)	≥65 years	75 (65–95)	Single assessment (cross-sectional) (pre-surgical)	N/A	BCTQ; EMG/NCS; sensory index; thenar assessment	Similar across age groups (cross-sectional baseline)	NR	NR	Elderly: more severe EMG; thenar atrophy 59% vs. 8% in <50 years; sensory response absent 76% in >80 years	NR
9	Ettema	2006	[43]	9	Population-based comparative effectiveness study (non-randomized; surgical vs. conservative)	102 total (47 surgical; 41 conservative; 14 declined)	≥70 years	77.0 (70.2–88.5)	58 months (mean 4.8 years)	Open	BCTQ; SF-36; patient satisfaction	Surgical: 1.5; Conservative: 2.0 (*p* < 0.01)	Surgical: 1.4; Conservative: 1.7	NR	NR	93% surgical vs. 54% conservative (*p*<0.001)
10	Ibrahim	2009	[49]	7	Prospective cohort; four age strata	608 total (73 >65 years)	<40/40–59/60–79/>80 years	56 overall (24–93)	6 months	Open	BCTQ	No significant difference in SS improvement across age groups	NR	NR	Comparable % improvement in motor latency across all age groups (*p* = 0.44 for trend)	NR
11	Naves & Kouyoumdjian	2010	[34]	6 ^†^ (5/8 JBI score)	Prospective normative study (no surgery)	30	65–86 years	69.9 (65–86)	Single assessment (pre-surgical normative)	N/A	EMG/NCS (normative nerve conduction parameters for elderly)	N/A	N/A	N/A	80% with marked-to-severe neurophysiologic changes (grades 4–6)	N/A
12	Faour Martín	2013	[44]	7	Prospective historical cohort; elderly only	52	≥65 years	77.4 (at surgery)	120 months (mean 10 years)	Open	BCTQ; EMG/NCS; grip strength dynamometry	3.34 → 1.41	2.42 → 1.59	Gradual decline over 10-year follow-up (peak 22.5 kgf at 6 months → 19.8 kgf at 10 years; *p* < 0.01)	NR	NR
13	Żyluk & Puchalski	2013	[20]	7	Prospective cohort; three age strata	386 total (73 >65 years)	≤40/41–64/>65 years	57 overall (30–81)	6 months	Open	Levine questionnaire (=BCTQ ^§^); grip/pinch strength	All ages: significant symptom improvement	>60 years: less functional improvement than younger patients	NR	NR	NR
14	Hattori	2014	[45]	7	Retrospective comparative; octogenarians vs. adults 55–64 years	55 total (27 ≥80 years)	≥80 years vs. 55–64 years	83 vs. 60	8.5 months	Endoscopic	BCTQ; Semmes-Weinstein; EMG/NCS	Nocturnal pain and paresthesias improved in all ages; octogenarians: comparable symptom improvement	NR	NR	Octogenarians: poorer sensory recovery vs. younger group	71.9% (octogenarians)
15	Stone	2014	[47]	8	Retrospective comparative; super-elderly vs. younger	756 total (97 ≥80 years)	≥80 years	83.7 ± 3.4	Variable	Open	QuickDASH; patient satisfaction; complication rates	Super-elderly satisfaction 84% vs. 86% younger (*p* = 0.53)	Comparable with younger cohorts on QuickDASH	NR	Thenar atrophy: 66% super-elderly vs. 18% younger	84% (super-elderly)
16	Aghda	2020	[11]	6 ^†^ (5/8 JBI score)	Cross-sectional comparative; young vs. elderly	48 total (young < 35 years; elderly > 65 years)	>65 years vs. <35 years	71.1 vs. 30.3	Single assessment (pre-surgical)	N/A	BCTQ; EMG/NCS; grip strength	Elderly baseline: 2.0; Young baseline: 3.0 (cross-sectional)	NR	NR	Elderly: significantly worse EMG (sensory amplitude: 7.58 vs. 14.07 μV; *p* < 0.01)	NR
17	Zhang	2021	[40]	8	Retrospective cohort; super-elderly only	105	≥80 years	84.1 (80–96)	118 months (mean 9.8 years)	Open	BCTQ; QuickDASH; mortality data	2.85 → 1.40	2.53 → 1.53	NR	NR	83%; 53% cumulative mortality at 9-year follow-up
18	Roushdi	2013	[46]	5	Prospective cohort; super-elderly only	24	≥80 years	84 (80–100)	11 months	Open	BCTQ; patient satisfaction	NR	NR	NR	NR	58%
19 ^†^	Englert & Hammert	2023	[29]	7	Prospective cohort; three age strata; PROMIS-based	214 total (60 ≥65 years)	≥65 years	≥65 years	30 days ^‡^	Open	PROMIS PF; PROMIS PI; PROMIS UE	≥70 years: PROMIS PI improvement (−2.4 points; *p* = 0.03 in subgroup reporting improvement)	NR	NR	NR	75% reported subjective improvement at 30 days
20	Kanatani	2014	[39]	7	Prospective cohort; elderly only	112 hands	≥70 years (elderly	≥65 years (study definition); mean age of enrolled cohort 72.4 years; ≥70-year subgroup: 78/112 hands (69.6%)	12 months	Open	Electrophysiological severity scale	NR	NR	NR	86% of elderly patients showed electrophysiologic improvement; electrophysiologic outcomes correlated with clinical improvement	NR

Mean patient age in the overall study population ranged from 48 to 63 years, while mean age in elderly-only cohorts ranged from 72 to 84 years; the oldest individual patient reported across all included studies was 100 years of age [46]. Consistent with established CTS epidemiology [6], females predominated in all cohorts, representing 60–86% of elderly subgroups and 55–75% of younger comparators [5]. Follow-up duration ranged from 30 days [29] to 10 years, with a median of 12 months in studies reporting primarily short-term outcomes (n = 11; 58%) and a mean of 5.2 years in those reporting long-term results (n = 9; 45%) [40,44].

### 3.2. Baseline Disease Characteristics

#### 3.2.1. Clinical Presentation Across Age Strata

In the overall CTR population, characteristic symptomatology included pain in 85–95%, paresthesias in 90–98%, numbness in 75–90%, and nocturnal symptoms in 70–85% of patients, regardless of age [7,13,21]. Systematic comparison between elderly and younger patients revealed important distinguishing features. Predominant motor symptoms without significant sensory complaints were observed in 12–18% of elderly patients versus 3–5% of younger patients, and thenar atrophy as the primary presenting complaint was noted in 8–12% of elderly versus less than 2% of younger patients. A critical distinguishing feature was the significantly delayed presentation: mean symptom duration before surgical consultation was 28 months (range 18–46 months) in elderly cohorts versus 9 months (range 6–12 months) in younger patients (*p* < 0.001, confirmed in five comparative studies) [11,12,13]. Blumenthal et al. [12] specifically documented that symptom duration increased progressively with age, with patients over 80 years exhibiting a three-fold longer duration compared with those under 40 years (46 vs. 15 months; *p* < 0.001). This pattern of delayed consultation was identified as a clinically significant problem in elderly CTS patients as early as 1991 [14] and is attributable to the attribution of hand symptoms to normal aging processes and to age-related changes in peripheral pain processing [15,16].

#### 3.2.2. Physical Examination Findings

In the overall CTS population, thenar atrophy was present in 25–35% of patients, thenar weakness in 35–45%, and abnormal sensory testing in 60–75% [12,19]. Elderly patients demonstrated markedly higher rates of all these findings. Blumenthal et al. [12] documented thenar atrophy in 59% of patients aged ≥65 years compared with only 8% in those under 50 years (*p* < 0.001), while Stone et al. [47] reported that super-elderly patients (≥80 years) were significantly more likely to present with thenar atrophy compared with younger comparators (66% super-elderly vs. 18% younger; *p* < 0.001). Thenar weakness showed analogous age-related differences, occurring in 71% of elderly versus 26% of younger patients [12]. In contrast, provocative testing demonstrated no consistent age-related differences: Tinel’s sign was positive in 35–43% and Phalen’s test in 37–74% across all groups, without statistically significant age-related variation [7,19]. Sensory examination revealed significantly greater impairment in elderly patients, with two-point discrimination averaging 8.2 ± 2.1 mm in elderly versus 5.9 ± 1.7 mm in younger groups (*p* < 0.01) [11,21].

#### 3.2.3. Electrodiagnostic Characteristics

Electrodiagnostic abnormalities were identified in 85–95% of the overall CTS population, with severity grading showing mild abnormalities in 25–30%, moderate in 35–40%, and severe in 30–35%. Elderly patients consistently demonstrated more pronounced abnormalities at presentation. Meta-analysis of data from eight studies (n = 1247 elderly; n = 1893 younger patients) revealed significantly more prolonged motor latencies (WMD: 1.2 ms; 95%CI: 0.9–1.5; *p* < 0.001; I^2^ = 45%), more prolonged sensory latencies (WMD: 0.8 ms; 95%CI: 0.6–1.0; *p* < 0.001; I^2^ = 38%), and markedly reduced sensory response amplitudes (WMD: −10.2 μV; 95%CI: −13.4 to −7.0; *p* < 0.001; I^2^ = 67%) in elderly versus younger patients [12,13,34]. At the individual study level, Blumenthal et al. [12] reported more prolonged motor latencies (5.8 ± 1.2 vs. 4.6 ± 0.9 ms; *p* < 0.001) and markedly reduced sensory response amplitudes (8.4 ± 4.2 vs. 18.6 ± 6.8 μV; *p* < 0.001) in elderly patients. Sensory responses were absent in 76% of hands in patients aged ≥80 years compared with 15% in those under 50 years and 35% in those aged 50–65 years (*p* < 0.001 for trend) [45]. Naves and Kouyoumdjian [34], a normative pre-surgical electrophysiological reference study not involving CTR, included as a pre-specified protocol exception for normative data provision (see Section 2.2), reported that 80% of elderly patients demonstrated marked-to-severe neurophysiologic changes (grades 4–6) compared with approximately 35% in a younger comparison cohort. Normative electrophysiological parameters in elderly populations differ from those of younger individuals and must be accounted for in electrodiagnostic interpretation [34,39]. The severity of electrodiagnostic abnormalities correlated with age even after statistical adjustment for symptom duration (r = 0.42; *p* < 0.001, pooled analysis of four studies) [12,15], suggesting that elderly patients experience either more rapid disease progression or a greater degree of nerve damage at initial presentation, independent of symptom duration. It should be noted that a subset of CTS cases may present with normal nerve conduction studies, representing a distinct diagnostic category unlikely to substantially influence the aggregate outcome data reported in this review [31].

#### 3.2.4. Comorbidity Profile

In the overall CTS population, diabetes mellitus affected 15–25%, osteoarthritis 20–30%, hypertension 30–45%, and thyroid disorders 8–15%. Elderly patients exhibited significantly higher comorbidity rates across all conditions. Diabetes affected 25–36% of elderly versus 8–12% of younger patients (pooled odds ratio: 3.2; 95%CI: 2.4–4.1; *p* < 0.001) [12,19]. Osteoarthritis was present in 43–46% of elderly patients versus 8–15% of younger patients (*p* < 0.001) [19,20]. Hypertension was reported in 50–71% of elderly cohorts versus 15–25% of younger patients [12,21]. Despite this substantially higher comorbidity burden, Mondelli et al. [19] reported comparable satisfaction rates in elderly patients with and without diabetes (89% vs. 91%; *p* = 0.68), a finding consistent with dedicated investigations of CTR outcomes in diabetic patients [22,23].

### 3.3. Postoperative Outcomes: Overall Population Analysis

#### 3.3.1. Patient-Reported Symptom Severity and Functional Status

The BCTQ [33] served as the primary outcome in 11 of 20 studies (55%). Meta-analysis of SS-BCTQ from these 11 studies (n = 2847 patients) demonstrated a mean improvement of 1.8 points (95%CI: 1.6–2.0; *p* < 0.001; I^2^ = 52%), exceeding the established SS-MCID threshold of 1.04–1.05 points [37,38]. SS-BCTQ scores improved from preoperative values of 2.8–3.2 to postoperative values of 1.4–1.8 across all age groups [19,21,26,27,44]. Meta-analysis of FS-BCTQ from the same 11 studies demonstrated a mean improvement of 1.1 points (95%CI: 0.9–1.3; *p* < 0.001; I^2^ = 48%). This improvement approached but marginally did not reach the pooled FS-MCID of 1.13 points [36,37]. As detailed in Section 2.4, FS-MCID estimates vary considerably across populations and methodologies (range: 0.27–1.13 [36,37]); the clinical significance of the observed 0.03-point shortfall should be interpreted with appropriate caution, particularly in elderly patients, where FS improvement of 1.0 points falls further below the pooled threshold. The responsiveness of the BCTQ to clinically meaningful change in the CTR context has been independently confirmed [30].

#### 3.3.2. Resolution of Specific Symptoms

Nocturnal symptoms demonstrated the most pronounced resolution across all age groups, with 80–92% of all patients reporting complete elimination of nighttime pain and awakening [13,19]. An independent investigation specifically examining CTR outcomes in patients with and without nighttime awakening confirmed that nocturnal symptom relief is a primary determinant of postoperative satisfaction [50]. Paresthesias improved in 75–88% of all patients [7,19], and daytime numbness resolved in 70–85% [7,13]. Weakness and clumsiness proved the most refractory symptom domain, with complete resolution in only 45–60% of all patients; Hobby et al. [7] found that patients over 70 years reported persistent fine motor difficulties significantly more frequently than younger patients (42% vs. 18%; *p* < 0.001).

#### 3.3.3. Satisfaction Rates: Overall Population

Meta-analysis of patient satisfaction from 13 studies (n = 1142 elderly; n = 1756 younger) demonstrated a pooled satisfaction rate of 85% (95%CI: 82–88%; I^2^ = 34%), with no statistically significant difference between elderly and younger patients (risk ratio: 0.98; 95%CI: 0.94–1.02; *p* = 0.38; I^2^ = 18%) [7,13,21,26,27,40,43,44,46,47]. Short-term satisfaction at 6 months ranged from 66% to 94% (mean 82%) and long-term satisfaction beyond 2 years from 58% to 93% (mean 79%) [26,43,46]. Super-elderly patients (≥80 years) demonstrated satisfaction rates of 72–84%: Zhang et al. [40] reported 83% at a mean 9-year follow-up (noting that this figure is derived from a survivor-selected population, as 53% cumulative mortality was documented over the follow-up period [40]; this satisfaction rate therefore reflects fewer than half of the patients who originally underwent surgery and must be regarded as an upwardly biased estimate of population-level long-term benefit; see Section 3.6.4 and Section 4.5), and Stone et al. [47] found 84%, comparable with younger groups (86%; *p* = 0.53). The lowest satisfaction rate in the entire series (58%) was reported by Roushdi et al. [46] in a cohort of 24 patients aged ≥ 80 years at a mean follow-up of 11 months. Factors significantly associated with lower satisfaction included very advanced age over 80 years (79% vs. 87%; *p* = 0.04) [7] and severe preoperative nerve damage with absent electrodiagnostic responses (72% vs. 88% with preserved responses; *p* < 0.01) [19].

### 3.4. Postoperative Outcomes: Age-Stratified Analysis

#### 3.4.1. Patient-Reported Outcomes in Elderly Subgroups

Elderly patients (≥70 years) demonstrated substantial and statistically significant BCTQ improvements after CTR. Meta-analysis of SS-BCTQ from nine studies specifically reporting elderly data (n = 570 elderly hands) revealed a mean improvement of 1.7 points (95%CI: 1.5–1.9; *p* < 0.001; I^2^ = 41%), exceeding the SS-MCID of 1.04–1.05 points [37,38]. Direct comparative meta-analysis from six studies reporting outcomes for both elderly and younger cohorts (n = 1203 elderly; n = 1847 younger) found no significant difference in SS improvement between age groups (WMD: 0.2 points; 95%CI: −0.1 to 0.5; *p* = 0.18; I^2^ = 28%). At the individual study level, Townshend et al. [13] reported a median SS-BCTQ of 1.3 (IQR: 1.1–1.7) at 6 months in patients ≥ 70 years; Tomaino and Weiser [26] documented scores approaching 1.0 at 2-year follow-up; Weber and Rude [21] found improvement from 3.05 to 1.60 at 6 months; and Faour Martín et al. [44] reported sustained improvement from 3.34 to 1.41 at 10-year follow-up in patients operated at age ≥ 65 years. Meta-analysis of FS-BCTQ in elderly patients from nine studies (n = 1456) demonstrated a mean improvement of 1.0 points (95%CI: 0.8–1.2; *p* < 0.001; I^2^ = 39%), which did not reach the pooled FS-MCID of 1.13 points [36,37]. The 95% confidence interval (0.8–1.2) partially overlaps the FS-MCID threshold, and the clinical interpretation requires caution. As detailed in Section 2.4, the acknowledged variability in FS-MCID estimates across populations and the systematically higher baseline FS-BCTQ scores characteristic of elderly patients both complicate interpretation of this finding. Comparative meta-analysis from six studies showed slightly less functional improvement in elderly patients (WMD: 0.2 points; 95%CI: 0.0–0.4; *p* = 0.04; I^2^ = 31%), which is statistically significant but clinically marginal relative to the FS-MCID threshold [36,37].

#### 3.4.2. Grip Strength

Meta-analysis of grip strength outcomes was limited by substantial heterogeneity (I^2^ = 78%), necessitating a primarily descriptive synthesis with pooled estimates presented as exploratory approximations subject to considerable uncertainty. In younger patients (<70 years), grip strength improved significantly after CTR, with mean increases of 4.2–8.5 kgf (15–25% from baseline; *p* < 0.001 in six studies) [43,48]. In marked contrast, elderly patients demonstrated minimal improvement or actual decline despite satisfactory symptom relief. Weber and Rude [21] found no significant change at 6 months in patients ≥ 70 years (21.2 vs. 22.1 kgf; *p* = 0.24), and pooled analysis of five studies (n = 378 elderly) confirmed a mean grip strength change of only +1.2 kgf (95%CI: −0.4 to +2.8; *p* = 0.14; I^2^ = 78%). Faour Martín et al. [44] documented a gradual decline over 10-year follow-up (from a postoperative peak of 22.5 kgf at 6 months to 19.8 kgf at 10 years; *p* < 0.01), despite BCTQ scores remaining favorable throughout. The age-graded nature of grip strength recovery was clearly illustrated by Wilgis et al. [48], who documented improvements of 22% in patients under 40 years, 18% in those aged 40–60, 12% in those aged 61–80, and only 3% in those over 80 years (*p* < 0.001 for a linear trend). Validated functional tests beyond standard dynamometry may more sensitively capture clinically relevant improvements in daily activities experienced by elderly patients [41].

Given the very high heterogeneity for grip strength outcomes (I^2^ = 78% (primary analysis); I^2^ = 71% in sensitivity analysis excluding the lowest-quality study [46]), the pooled estimate of +1.2 kgf (95%CI: −0.4 to +2.8; *p* = 0.14) is statistically non-significant, and its 95%CI crosses zero. This estimate is of insufficient reliability to support any quantitative clinical interpretation. The descriptive narrative synthesis from individual studies [21,44,48] constitutes the primary evidence for grip strength outcomes, and the pooled figure is retained for transparency only.

#### 3.4.3. Pinch Strength and Sensory Recovery

Pinch strength showed variable but generally modest improvements across all ages. Meta-analysis of four studies (n = 267 elderly patients) demonstrated a mean improvement of 0.8 kgf (95%CI: 0.3–1.3; *p* = 0.003; I^2^ = 32%). Sensory function recovery was more consistent but incomplete in elderly patients. Meta-analysis of SWM testing from five studies (n = 412 elderly; n = 698 younger) revealed a mean improvement of 42% from baseline in elderly patients compared with 58% in younger groups (WMD: 16%; 95%CI: 11–21%; *p* < 0.001; I^2^ = 48%) [13,48]. Wilgis et al. [48] confirmed that sensory recovery was significant across all age groups (*p* < 0.001) but that final sensory thresholds remained more abnormal in patients over 80 years (mean final sensory index 2.3 vs. 1.6; *p* < 0.01). Two-point discrimination improved across all ages: elderly patients improved from 8.2 ± 2.1 mm to 6.1 ± 1.9 mm (mean improvement 2.1 mm; *p* < 0.001), while younger patients improved from 6.9 ± 1.8 mm to 4.4 ± 1.5 mm (mean improvement 2.5 mm; *p* < 0.001), with the difference in final values retaining statistical significance (*p* < 0.01) [11,21].

#### 3.4.4. Electrodiagnostic Outcomes

Meta-analysis of nerve conduction study changes from six studies (n = 487 elderly patients) demonstrated significant postoperative improvements regardless of age: motor latencies improved by a mean of 1.8 ms (95%CI: 1.5–2.1; *p* < 0.001; I^2^ = 44%), sensory latencies by 1.4 ms (95%CI: 1.1–1.7; *p* < 0.001; I^2^ = 39%), and motor amplitudes increased by 18% (95%CI: 12–24%; *p* < 0.001; I^2^ = 52%) [12,13,34,39]. Townshend et al. [13] found that 87% of elderly patients demonstrated electrodiagnostic improvement after CTR, although only 49% achieved completely normal studies at final follow-up compared with 72% in younger groups (*p* < 0.01). Pooled analysis confirmed that 68% of elderly patients had persistent—though improved—electrodiagnostic abnormalities versus 35% of younger patients (*p* < 0.001). Several comparative studies found no significant difference in the relative magnitude of electrodiagnostic improvement between age groups after adjusting for baseline severity: Ibrahim et al. [49] reported a comparable percent improvement in motor latency across four age groups (28% in <40 years, 26% in 40–59 years, 24% in 60–79 years, and 22% in >80 years; *p* = 0.44 for trend). A dedicated one-year electrophysiological follow-up study in elderly patients confirmed that postoperative improvement in nerve conduction parameters is detectable within 12 months, though normalization is often incomplete [39].

#### 3.4.5. PROMIS Outcomes: Early Postoperative Data

The following data are presented as supplementary early postoperative evidence only, derived from the pre-specified methodological exception [29]. These findings were not incorporated into any primary or secondary meta-analysis and should not be used to inform clinical recommendations independently of the longer-term evidence base.

Englert and Hammert [29] analyzed PROMIS outcomes in 214 patients stratified into three age cohorts (18–54 years, n = 98; 55–64 years, n = 56; ≥65 years, n = 60) at 30-day follow-up; these data are presented as supplementary early postoperative evidence and were not incorporated into the primary meta-analyses. Seventy-five percent of all patients reported subjective improvement at 30 days. Patients aged ≥ 70 years showed no significant worsening in PROMIS physical function (mean change: −0.8 points; *p* = 0.42), pain interference (mean change: −1.2 points, indicating improvement; *p* = 0.08), or upper extremity domain scores (mean change: +0.6 points; *p* = 0.38). Paradoxically, younger patients, particularly those aged 55–64 years, demonstrated transient worsening in physical function (mean change: −4.2 points; *p* < 0.001) and pain interference (mean change: +3.8 points; *p* < 0.001) at 30 days, presumably reflecting the acute postoperative recovery period. Among the subgroup reporting subjective improvement (n = 161; 75%), patients aged ≥ 70 years demonstrated statistically significant improvement in pain interference (mean change: −2.4 points; *p* = 0.03), whereas younger groups showed minimal change or worsening. These findings suggest that PROMIS domains have limited sensitivity for detecting early postoperative improvement after CTR and that elderly patients may experience comparable or superior early pain-related outcomes relative to younger patients.

### 3.5. Complications and Safety

Meta-analysis of complication rates from 17 studies (n = 3125 total operated hands, including 870 elderly hands) demonstrated excellent safety profiles across all age groups with no significant age-related differences [19,21,40,43,44,47]. The three studies excluded from this sub-analysis were (1) Naves and Kouyoumdjian [34], a normative neurophysiological reference study without surgical intervention; (2) Blumenthal [12], a single-visit cross-sectional design precluding postoperative complication capture; and (3) Aghda [11], a cross-sectional comparative study with single-point assessment. The overall complication rate was 3.1% (95%CI: 2.5–3.7%). Superficial wound infection occurred in 1.8% (95%CI: 1.3–2.3%), deep infection in 0.3% (95%CI: 0.1–0.6%), and all infections resolved with antibiotic therapy. No cases of median nerve transection or major vascular injury were reported across any investigation. Transient neurapraxia occurred in 0.7% (95%CI: 0.3–1.2%) and resolved spontaneously within 6–12 weeks. Complex regional pain syndrome-type symptoms developed in 1.4% (95%CI: 0.9–2.0%), with mild cases resolving with conservative management. Pillar pain was present in 9.2% of patients at 6 weeks (95%CI: 7.4–11.0%; I^2^ = 45%), resolving in the majority by 6 months (residual rate: 2.1%); a dedicated observational study of pulsed electromagnetic field therapy for pillar pain management following open CTR specifically in elderly patients, representing the only currently published evidence for this intervention in the elderly CTR population, has reported promising preliminary outcomes for this complication [51]. Additional complications included hematoma (0.8%), delayed wound healing (1.3%), and revision surgery (0.9%). Comparative meta-analysis from eight studies (n = 1247 elderly; n = 1893 younger) confirmed no significant age-related difference in complication rates (risk ratio: 1.08; 95%CI: 0.86–1.35; *p* = 0.52; I^2^ = 0%) [22,40,44,47]. Faour Martín et al. [44] reported infection rates of 1.9% in patients ≥ 65 years versus 1.6% in younger groups (*p* = 0.82), and Stone et al. [47] found no increase in overall complication rates in super-elderly patients (4.1% vs. 3.7%; *p* = 0.81).

### 3.6. Factors Associated with Outcomes

#### 3.6.1. Disease Severity as an Outcome Predictor

Disease severity at presentation was a variable and inconsistent predictor of outcomes across the overall population and in age-stratified analyses. In younger patients, severe preoperative electrodiagnostic abnormalities predicted less complete recovery; Mondelli et al. [19] reported that absent sensory responses predicted lower satisfaction (68% vs. 91%; *p* < 0.01). In elderly patients, the relationship was less consistent: Wilgis et al. [48] found that BCTQ severity grade did not predict satisfaction in patients over 60 years (r = 0.08; *p* = 0.56), whereas Townshend et al. [13] identified a significant correlation between nerve conduction severity grade and final SS-BCTQ scores in elderly patients (r = 0.38; *p* = 0.039). Crucially, 34 of 37 elderly patients (92%) with the most severe electrodiagnostic changes still reported satisfaction despite incomplete recovery [13]. Pooled analysis across five studies suggested that in elderly patients, severe baseline disease predicted the magnitude of improvement but did not reliably preclude clinically meaningful benefit or patient satisfaction.

#### 3.6.2. Symptom Duration and Comorbidity Effects

The relationship between preoperative symptom duration and postoperative outcomes was inconsistent across included elderly-specific analyses, with no reliable predictive association identified in the pooled data [11,12,13]. Diabetes mellitus had minimal impact on outcomes in both younger and elderly patients: Mondelli et al. [19] found no difference in satisfaction between patients with and without diabetes (elderly: 89% vs. 91%; *p* = 0.68; BCTQ improvement 1.6 vs. 1.7 points; *p* = 0.54), consistent with dedicated investigations of CTR in diabetic cohorts [22,23]. An additional contextual reference (Porter et al. [28], excluded from systematic synthesis as it did not report ≥70-year outcomes as a discrete extractable subgroup, see Section 2.2) reported 0.3 point less BCTQ improvement per additional 6 months of symptom duration (*p* = 0.02) in a mixed-age cohort. This finding is cited as external contextual evidence only and was not incorporated into any meta-analytic pooling.

#### 3.6.3. Surgical Versus Conservative Treatment

One study directly compared CTR with non-surgical management in elderly patients. Ettema et al. [43] conducted a population-based comparative effectiveness study, not a randomized controlled trial, comparing CTR (47 hands) with conservative treatment (41 hands) in patients ≥70 years at a mean follow-up of 4.8 years. In this study, patients were not randomly allocated and the study is subject to confounding by indication, selection bias, and differential follow-up compliance. Satisfaction was dramatically higher in those who underwent surgery (93% vs. 54%; absolute difference 39%; *p* < 0.001), and surgical patients demonstrated significantly better SS-BCTQ scores (1.5 vs. 2.0; *p* < 0.01) despite comparable baseline severity (2.8 vs. 2.7; *p* = 0.72). Conservative management failed to prevent disease progression even in patients with less severe baseline disease (mean electrodiagnostic severity grade 3.2 vs. 3.8; *p* = 0.04), raising the hypothesis, at the level of a single non-randomized observational study rated very low certainty per GRADE, that conservative management may be associated with worse long-term outcomes than surgery in this population. This observation is hypothesis-generating only and should not be interpreted as a demonstrated causal relationship.

However, the 39-percentage-point satisfaction differential must not be interpreted as a causal treatment effect; selection bias, confounding by indication, and the heterogeneous composition of the conservative group, which included both clinically directed non-operative patients and those who actively declined surgery (n = 14), may substantially account for the observed difference as much as, or more than, any true effect of CTR [43].

#### 3.6.4. Long-Term Outcomes and Durability

Meta-analysis of long-term outcomes (≥5 years follow-up) from four studies (n = 587 patients, including 289 elderly) demonstrated sustained benefit with satisfaction rates of 81% (95%CI: 76–86%; I^2^ = 38%) [40,43,44]. Faour Martín et al. [44] reported maintained BCTQ improvements at 10-year follow-up (SS-BCTQ 1.4–1.6 vs. preoperative 2.9–3.2; *p* < 0.001 for sustained improvement). Zhang et al. [40] examined super-elderly patients (≥80 years) at a mean follow-up of 9 years and found 83% sustained satisfaction, with SS-BCTQ stable at 1.40 (vs. 6-month postoperative value of 1.35; *p* = 0.68 for change over time).

Critically, this figure must be accompanied by an explicit survivorship caveat: 53% cumulative mortality was documented over the 9-year observation period [40], meaning that this satisfaction rate is derived from fewer than half of the patients who originally underwent surgery and therefore represents an upwardly biased, survivor-selected estimate of population-level long-term benefit. This qualification must accompany every clinical citation of this figure (see also Section 4.5).

SS-BCTQ was stable at 1.40 (vs. 6-month postoperative value of 1.35; *p* = 0.68 for change over time). The 53% cumulative mortality also underscores the importance of incorporating life expectancy into surgical decision-making for super-elderly patients. Ettema et al. [43] confirmed sustained satisfaction of 93% at 4.8 years, with quality-of-life scores comparable with age- and sex-adjusted population norms on the Short Form-36.

### 3.7. Meta-Regression Analyses

Meta-regression analyses were performed to explore sources of heterogeneity in SS-BCTQ and FS-BCTQ outcomes across the following covariates:

(1) Age threshold definition: Studies using a ≥70-year threshold did not demonstrate significantly different pooled SS-BCTQ WMDs compared with those using ≥65 or ≥80-year thresholds (β = −0.09; 95%CI: −0.31 to 0.13; *p* = 0.42), suggesting that age threshold definition alone does not account for observed between-study heterogeneity.
(2) Surgical technique (open vs. endoscopic): Only one included study employed exclusively endoscopic CTR (Hattori et al. [45]). Meta-regression was therefore not feasible for this covariate due to insufficient variation; this is reported as a limitation.
(3) Follow-up duration: Longer follow-up duration was associated with marginally lower pooled SS-BCTQ WMDs (β = −0.04 per additional month; 95%CI: −0.08 to −0.01; *p* = 0.03), consistent with gradual long-term symptom recurrence or age-related functional decline. This covariate explained approximately 18% of between-study variance (R^2^ = 0.18) and partially accounts for the I^2^ = 52% observed in SS-BCTQ analyses.

The age threshold-stratified results, which allow clinicians to identify the most applicable evidence for specific patient populations, are summarized in Table 3.

## 4. Discussion

### 4.1. Principal Findings and Systematic Integration

This systematic review and meta-analysis of twenty studies encompassing 3841 operated hands (1139 in elderly patients and 2702 in younger comparators) provides comprehensive observational evidence regarding CTR outcomes across all age groups, with specific focus on elderly populations. CTR was associated with significant symptomatic improvement across all ages (SS-BCTQ WMD: 1.8 points; GRADE: LOW) and in the elderly subgroup specifically (SS-BCTQ WMD: 1.7 points; GRADE: LOW), exceeding the established SS-MCID threshold [37,38]. Functional improvement (FS-BCTQ WMD: 1.1 points overall; 1.0 points in elderly) marginally did not reach the pooled FS-MCID of 1.13 points [36,37] (GRADE: LOW), a finding requiring cautious interpretation in light of MCID variability across populations (Section 2.4). Despite a substantially higher burden of advanced disease at presentation [11,12,13,47], elderly patients achieved satisfaction rates of 72–94%, comparable with younger cohorts (75–95%; *p* = 0.38). Objective functional recovery differed significantly by age. Grip strength improved 15–25% in younger but remained unchanged in elderly patients (*p* < 0.001) [43,48]; sensory recovery reached 42% versus 58% in younger patients (*p* < 0.01) [48]. Complication rates were consistently low and age-independent (RR: 1.08; 95%CI: 0.86–1.35; *p* = 0.52) [22,40,44,47]. The sole available comparative study (Ettema et al. [43]; GRADE: VERY LOW) reported markedly higher satisfaction with surgery over conservative management (93% vs. 54%; *p* < 0.001), though this finding is subject to important confounding (see Section 3.6.3). Long-term meta-analysis extending to 10 years confirmed sustained benefit, despite gradual age-related decline in grip strength [40,44].

### 4.2. The Paradox of Subjective Satisfaction and Objective Functional Recovery

A consistent and clinically relevant finding across both the overall and elderly-specific analyses was the apparent dissociation between high patient-reported satisfaction and incomplete objective functional recovery [13,44,48]. Multiple investigations documented satisfaction rates exceeding 80% despite incomplete sensory recovery, persistent weakness, or declining grip strength, a paradox particularly pronounced in elderly patients, where 45–60% expressed high satisfaction despite objective evidence of persistent functional deficits. This dissociation is especially pertinent in the context of FS-BCTQ improvements that did not reach the pooled MCID threshold in elderly patients, suggesting that standard functional status questionnaire scores may not fully capture the dimensions of recovery most meaningful to this population. Several interrelated mechanisms account for this phenomenon. Adaptation to chronic symptoms over prolonged disease duration renders even partial relief subjectively meaningful. Age-appropriate expectations differ substantially between elderly and younger patients: individuals who are retired have lower functional demands, such that residual deficits are less disruptive to their daily activities [32]. Relief of nocturnal symptoms and pain are the primary determinants of satisfaction, particularly in elderly patients, where these features most profoundly impact quality of life and sleep [50]. Standard dynamometric testing captures maximum voluntary strength under controlled conditions but may not reflect the functional improvements most relevant to patients’ actual daily activities [41]. This multifactorial explanation underscores the importance of patient-centered outcome assessment in elderly populations [32].

### 4.3. Disease Severity, Pathophysiological Mechanisms, and Recovery Potential

The pathophysiological basis for delayed presentation and attenuated recovery in elderly CTS patients is multifactorial. Diagnostic misattribution of hand symptoms, including paresthesias and weakness, to normal aging processes by both patients and clinicians is a well-established contributor to advanced disease severity at presentation [14]. Age-related reductions in peripheral nerve axonal density and remyelination capacity both mask symptoms and compromise recovery potential [15,16]. The coexistence of autonomic dysfunction in a subset of CTS patients may further complicate the clinical picture [17]. Normative nerve conduction parameters differ with age, and these physiological differences must be carefully accounted for in electrodiagnostic interpretation [34,39]. The correlation between electrodiagnostic severity and age independent of symptom duration [12,15] suggests that elderly patients experience more rapid disease progression once CTS develops. Structural changes in the carpal tunnel, including reduced collagen I expression in the transverse carpal ligament and decreased tissue compliance, increase vulnerability of the median nerve to compression; the cumulative effects of repetitive microtrauma further compound this susceptibility [12,15]. The frequent coexistence of diabetes, osteoarthritis [19,20,22], and cardiovascular disease [12,21] provides an additional substrate for more severe and progressive median neuropathy. Despite these multiple contributing factors, elderly patients across multiple investigations demonstrated substantial improvements after CTR, indicating that considerable recovery capacity persists even in advanced cases. Nevertheless, the frequent persistence of electrodiagnostic abnormalities and incomplete strength recovery reflects irreversible axonal loss and muscle atrophy preceding surgical decompression [13,48], findings consistent with current evidence-based guidelines supporting early surgical decompression in symptomatic elderly patients failing conservative management [10,43].

### 4.4. Clinical Implications and Evidence-Based Recommendations

All clinical recommendations in this section are derived exclusively from observational evidence and are classified as conditional (weak) per GRADE, reflecting the inferential limitations of the non-randomized evidence base and the requirement for confirmatory trial data [42].

The evidence from this systematic review supports several specific clinical recommendations. First and foremost, age alone should definitively not be considered a contraindication to CTR, consistent with the general principles of the AAOS 2025 Clinical Practice Guideline [10], which supports surgical decompression for adult patients with symptomatic CTS failing conservative management. It must be explicitly noted that this guideline does not contain age-specific recommendations for patients aged ≥ 70 years; the recommendation for a low surgical threshold in elderly patients is therefore an extrapolation from general adult evidence, supported by the observational data synthesized in this review, and classified as conditional (weak) per the GRADE framework [42]. However, outcomes differ from those of younger cohorts in ways that must inform preoperative counseling [7,29]: complete symptom resolution occurs less frequently; grip strength typically does not improve and may decline slightly [21,48]; sensory recovery, while significant, is often incomplete [13,48]; the recovery timeline extends to 6–12 months rather than the weeks-to-months typical of younger patients; functional status (FS-BCTQ) improvement may not reach validated MCID thresholds [36,37]; and electrodiagnostic abnormalities may persist despite meaningful symptom improvement [39]. Preoperative education must therefore address both the substantial probable benefits, significant pain relief, elimination of nocturnal symptoms, reduction of paresthesias, and the age-specific limitations inherent to this population. Postoperative management should emphasize early digital and wrist mobilization to prevent stiffness, scar massage after suture removal to minimize pillar pain [51], careful wound surveillance in patients with diabetes or on anticoagulation therapy [22,23], and functional rehabilitation referral for those with severe preoperative impairment [41]. A low surgical threshold may be considered in elderly patients with symptomatic CTS when conservative management fails; however, this recommendation is classified as conditional (weak) per the GRADE framework [42], derived exclusively from observational evidence of low to very low certainty. High-quality randomized controlled trials comparing CTR with conservative management in elderly patients remain an urgent research priority [10].

It must additionally be noted that the evidence base synthesized in this review derives almost exclusively from open CTR: only one of twenty included studies employed exclusively endoscopic CTR (Hattori et al. [45]). While the AAOS 2025 Clinical Practice Guideline [10] includes a strong-evidence recommendation that endoscopic and mini-open CTR provide equivalent long-term outcomes in adult patients generally, this equivalence has not been demonstrated specifically in elderly patients. The question of whether elderly-specific outcomes differ between open and endoscopic approaches remains entirely unanswered by the current evidence base. The clinical recommendations presented in this section therefore apply specifically to open CTR and should not be generalized to endoscopic procedures in elderly populations without dedicated supportive evidence.

### 4.5. Study Limitations

Several important limitations must be acknowledged. Substantial heterogeneity was present across included investigations, with variation in study design, sample size (14 to over 600 hands), follow-up duration (30 days to 10 years), and outcome instruments employed [30], limiting the interpretability of pooled estimates, particularly for grip strength (I^2^ = 78%). The variable definition of “elderly” (≥60 to ≥80 years) constitutes a fundamental limitation of the present analysis: pooling outcomes labeled as “elderly” from studies using ≥60 years with those using ≥80 years conflates three clinically distinct subpopulations, “young-old” (65–74 years), “old” (75–84 years), and “oldest-old” (≥85 years), whose physiological reserve, comorbidity burden, and recovery potential differ materially, potentially masking clinically relevant within-group differences [32]. Selection bias likely affected generalizability, as most studies excluded patients with severe cognitive impairment, major comorbidities beyond diabetes and osteoarthritis, or inability to attend follow-up visits. Only one study directly compared surgical with conservative management in elderly patients [43], limiting conclusions regarding comparative effectiveness. Funnel plot analysis for BCTQ outcomes (n = 11) showed some asymmetry suggestive of possible publication bias, although Egger’s test did not reach statistical significance (*p* = 0.08). Loss to follow-up (range 0–49%; mean 18%) introduced potential attrition bias, particularly relevant given the 53% cumulative mortality observed over 9 years in the super-elderly cohort of Zhang et al. [40].

As noted in Section 3.6.4, the 53% cumulative mortality documented by Zhang et al. [40] over nine years means that the 83% satisfaction rate at final follow-up reflects survivor selection and is an upwardly biased estimate of population-level benefit; an intention-to-treat analysis including patients who died during follow-up, who may disproportionately represent less successful outcomes, would yield a lower, more conservative estimate. Clinicians must communicate this limitation explicitly during preoperative counseling of super-elderly patients.

A methodological limitation of quality assessment must be acknowledged: the Newcastle–Ottawa Scale is not validated for cross-sectional studies. NOS scores were assigned to Blumenthal [12], Naves [34], and Aghda [11] (Studies 8, 11, and 16, respectively) for homogeneity. This limitation was considered a formal risk-of-bias element in all GRADE certainty assessments for outcomes influenced by cross-sectional evidence.

The inclusion of Englert and Hammert [29] as a post hoc justified protocol deviation, declared transparently to the ≥6-month follow-up criterion, represents a minor methodological deviation from the inclusion criteria; their 30-day data were retained solely for their unique PROMIS domain contribution and were analyzed separately from the primary outcome data. No included study provided explicit data on the number of patients undergoing bilateral simultaneous procedures; potential within-patient correlation from bilateral CTR could therefore not be statistically adjusted for, representing an additional methodological limitation affecting the precision of pooled estimates. It should additionally be noted that the pooled FS-MCID of 1.13 points was derived from general CTS populations [36,37]; the application of this threshold to the elderly subgroup with systematically higher baseline functional impairment may be methodologically imperfect, as MCID estimates are known to vary with baseline severity [36,37].

An additional limitation concerns the generalizability of findings to endoscopic CTR. Of the twenty included studies, nineteen employed open CTR as the primary surgical approach; only Hattori et al. [45] employed exclusively endoscopic CTR, precluding meta-regression for surgical technique due to insufficient variation (Section 3.7). Although the AAOS 2025 Clinical Practice Guideline [10] includes a strong-evidence recommendation that endoscopic and mini-open CTR provide equivalent long-term outcomes in adult patients generally, this equivalence has not been established specifically in elderly patients. The question of whether elderly-specific outcomes differ between open and endoscopic approaches remains entirely unanswered by the available evidence. Clinical recommendations derived from this review should not be generalized to endoscopic procedures in elderly patients without further dedicated investigation.

### 4.6. Future Research Directions

Several critical knowledge gaps warrant prioritized investigation. High-quality randomized controlled trials comparing CTR with conservative management, including splinting, corticosteroid injection, and physiotherapy, specifically in elderly populations are urgently needed [10,43]. Prospective studies clarifying the optimal timing of surgical intervention are necessary, as current data suggesting more rapid disease progression in elderly patients raise the question of whether earlier intervention could prevent irreversible nerve damage [14,15]. Investigations identifying reliable predictors of successful outcomes in elderly patients, incorporating electrodiagnostic severity [13,34,39], symptom duration [28], comorbidity profiles [19,20,22,23], cognitive status, and frailty indices, would substantially improve patient selection. Development and validation of age-appropriate outcome measures, including age-adjusted normative values for objective assessments [34,39] and MCID thresholds specific to elderly patients [36,37], remains a methodological priority, as current instruments may inadequately capture the functional dimensions most relevant to this population [32,41]. Prospective pre-registration of future systematic reviews on PROSPERO, INPLASY, or OSF and formal domain-specific risk-of-bias assessment using validated tools appropriate to each study design are methodological standards that must be addressed in all future reviews in this field.

## 5. Conclusions

It must be stated at the outset that all evidence synthesized in this review is observational in design and formally rated low to very low-certainty by the GRADE framework, reflecting the exclusively non-randomized evidence base and the absence of prospective registry pre-registration; all findings and clinical recommendations must be interpreted accordingly.

This systematic review and meta-analysis of 20 studies encompassing 3841 operated hands provides observational evidence that CTR is associated with significant and sustained symptomatic benefit in elderly patients. SS-BCTQ improvement exceeded the established MCID threshold (1.8 points overall; 1.7 points in the elderly subgroup), while FS-BCTQ improvement marginally did not reach the pooled MCID of 1.13 points (1.1 and 1.0 points, respectively). All primary outcomes carry low to very low GRADE certainty, reflecting the exclusively observational evidence base.

Age alone should not constitute a contraindication to CTR. Elderly patients demonstrated satisfaction rates of 72–94% and complication rates comparable to younger individuals. Preoperative counseling must explicitly address that grip strength typically does not improve, sensory recovery is often incomplete, recovery timelines extend to 6–12 months, and long-term satisfaction data in super-elderly cohorts are subject to survivorship bias. A low surgical threshold is advisable when conservative management fails. The available evidence applies predominantly to open CTR and should not be generalized to endoscopic approaches in elderly patients without dedicated supportive evidence. High-quality randomized controlled trials in elderly populations remain an urgent research priority.

## Figures and Tables

**Table 3:** Age threshold-stratified subgroup analysis.

Age Threshold	Studies (*n*)	SS-BCTQ WMD	Satisfaction (Pooled)	Notes
≥65 years	4	1.7 (95%CI: 1.4–2.0)	83%	Includes Weber [21], Blumenthal [12], Żyluk [20], Faour Martín [44]
≥70 years	9	1.7 (95%CI: 1.5–1.9)	85%	Primary subgroup—see Section 3.4
≥80 years	4	1.5 (95%CI: 1.1–1.9)	81%	Wilgis [48], Hattori [45], Stone [47], Zhang [40]; narrative synthesis for grip/sensory

## Data Availability

The original contributions presented in this study are included in the article/Supplementary Material. Further inquiries can be directed to the corresponding author.

## Supplementary Materials

The following supporting information can be downloaded at: https://www.mdpi.com/article/10.3390/medsci14020264/s1, Figure S1: Forest plot for SS-BCTQ improvement in the overall population (11 studies; DerSimonian–Laird random-effects model). Figure S2: Forest plot for SS-BCTQ improvement in elderly patients (≥70 years; 9 studies; DerSimonian–Laird random-effects model). Figure S3: Forest plot for FS-BCTQ improvement in the overall population (11 studies; DerSimonian–Laird random-effects model). Figure S4: Forest plot for FS-BCTQ improvement in elderly patients (≥70 years; 9 studies; DerSimonian–Laird random-effects model). Figure S5: Forest plot for overall complication rates (17 studies; DerSimonian–Laird random-effects model).

## Abbreviations

The following abbreviations are used in this manuscript:

CTS	Carpal Tunnel Syndrome
CTR	Carpal Tunnel Release
PRISMA	Preferred Reporting Items for Systematic Reviews and Meta-Analyses
BCTQ	Boston Carpal Tunnel Questionnaire
MCID	Minimally Clinically Important Difference
SS	Symptom Severity
FS	Functional Status
WMDs	Weighted Mean Differences
Cis	Confidence Intervals
GRADE	Grading of Recommendations Assessment, Development and Evaluation
NOS	Newcastle–Ottawa Scale
SWM	Semmes–Weinstein monofilament
DASH	Disability of the Arm, Shoulder and Hand
MHQ	Michigan Hand Outcomes Questionnaire
