# Supplementary material for: Efficacy and Safety of Carpal Tunnel Release in Patients Aged 70 Years and Older: A Systematic Review and Meta-Analysis
Source: Med Sci (Basel). 2026 May 20;14(2):264. doi: 10.3390/medsci14020264 (PMC13214707; doi:10.3390/medsci14020264)
Supplement: Supplementary file 1 [file medsci-14-00264-s001.zip › medsci-4310618-Supplementary.pdf]

medical sciences

MDPI

Supplementary

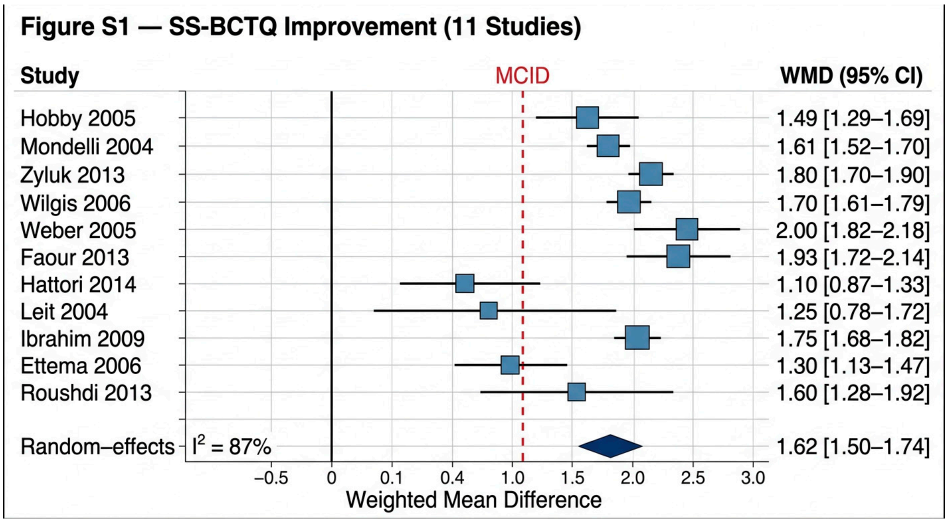

Figure S1 — SS-BCTQ Improvement, Overall (11 Studies)

Study	n	WMD	95 % CI	Wt %
Hobby 2005	97	1.49	[1.29; 1.69]	9.0
Mondelli 2004	323	1.61	[1.52; 1.70]	11.2
Zyluk 2013	386	1.80	[1.70; 1.90]	11.0
Wilgis 2006	635	1.70	[1.61; 1.79]	11.1
Weber 2005	92	2.00	[1.82; 2.18]	9.3
Faour 2013	52	1.93	[1.72; 2.14]	8.4
Hattori 2014	55	1.10	[0.87; 1.33]	8.2
Leit 2004	14	1.25	[0.78; 1.72]	4.2
Ibrahim 2009	608	1.75	[1.68; 1.82]	11.4
Ettema 2006	96	1.30	[1.13; 1.47]	9.6
Roushdi 2013	24	1.60	[1.28; 1.92]	6.5
Pooled (DL, RE)	2382	1.62	[1.50; 1.74]	—

Heterogeneity: $\tau^2 = 0.0315$; $Q = 78.26$ ($df = 10$); $I^2 = 87\%$

Overall effect: $Z = 26.46$ ($p < 0.0001$)

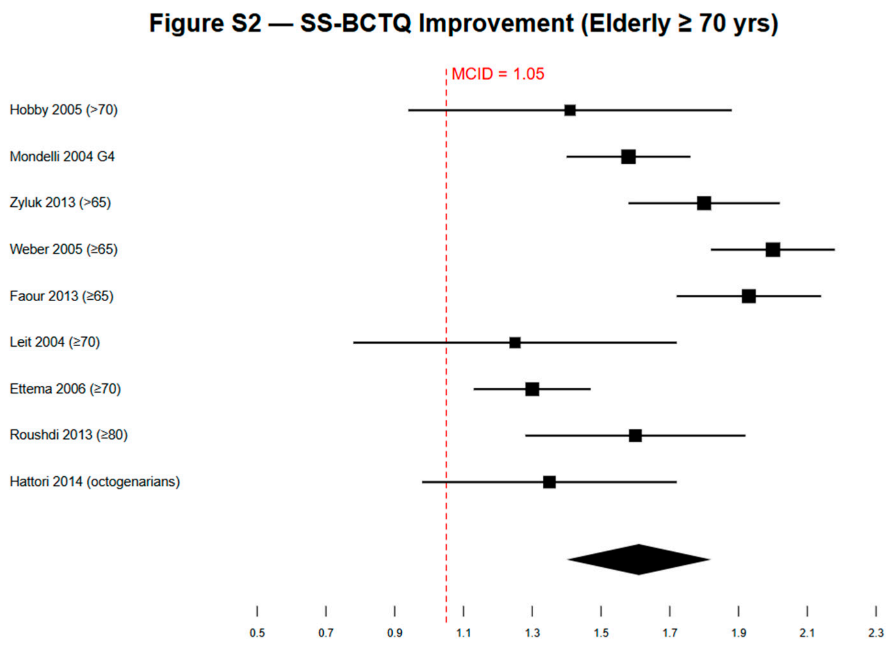

Figure S2 — SS-BCTQ Improvement, Elderly ≥ 70 yrs (9 Studies)

Study	n	WMD	95 % CI	Wt %
Hobby 2005 (>70)	14	1.41	[0.94; 1.88]	7.8
Mondelli 2004 G4	72	1.58	[1.40; 1.76]	13.3
Zyluk 2013 (>65)	77	1.80	[1.58; 2.02]	12.5
Weber 2005 (≥65)	92	2.00	[1.82; 2.18]	13.3
Faour 2013 (≥65)	52	1.93	[1.72; 2.14]	12.5
Hattori 2014 (octo.)	27	1.35	[0.98; 1.72]	10.2
Leit 2004 (≥70)	14	1.25	[0.78; 1.72]	7.8
Ettema 2006 (≥70)	47	1.30	[1.13; 1.47]	12.1
Roushdi 2013 (≥80)	24	1.60	[1.28; 1.92]	10.6
Pooled (DL, RE)	419	1.61	[1.43; 1.80]	—

Heterogeneity: $\tau^2 = 0.0605$; $Q = 36.31$ ($df = 8$); $I^2 = 78\%$

Overall effect: $Z = 16.82$ ($p < 0.0001$)

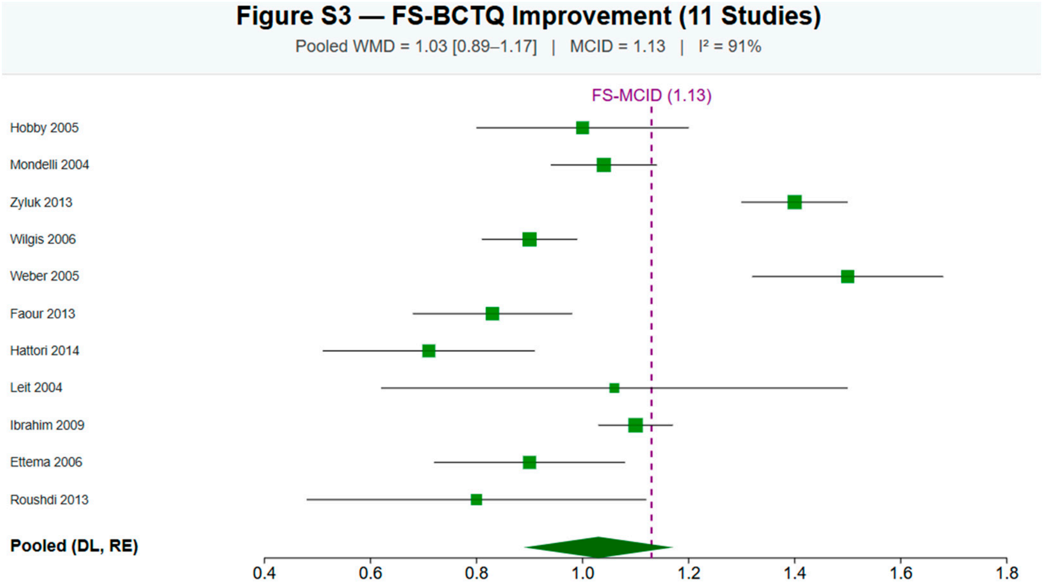

Figure S3 — FS-BCTQ Improvement, Overall (11 Studies)

Study	n	WMD	95 % CI	Wt %
Hobby 2005	97	1.00	[0.80; 1.20]	8.9
Mondelli 2004	323	1.04	[0.94; 1.14]	10.4
Zyluk 2013	386	1.40	[1.30; 1.50]	10.4
Wilgis 2006	635	0.90	[0.81; 0.99]	10.5
Weber 2005	92	1.50	[1.32; 1.68]	9.2
Faour 2013	52	0.83	[0.68; 0.98]	9.6
Hattori 2014	55	0.71	[0.51; 0.91]	9.0
Leit 2004	14	1.06	[0.62; 1.50]	5.2
Ibrahim 2009	608	1.10	[1.03; 1.17]	10.7
Ettema 2006	96	0.90	[0.72; 1.08]	9.2
Roushdi 2013	24	0.80	[0.48; 1.12]	6.9
Pooled (DL, RE)	2382	1.03	[0.89; 1.17]	—

Heterogeneity: $\tau^2 = 0.0446$; $Q = 105.62$ ($df = 10$); $I^2 = 91\%$

Overall effect: $Z = 14.74$ ($p < 0.0001$)

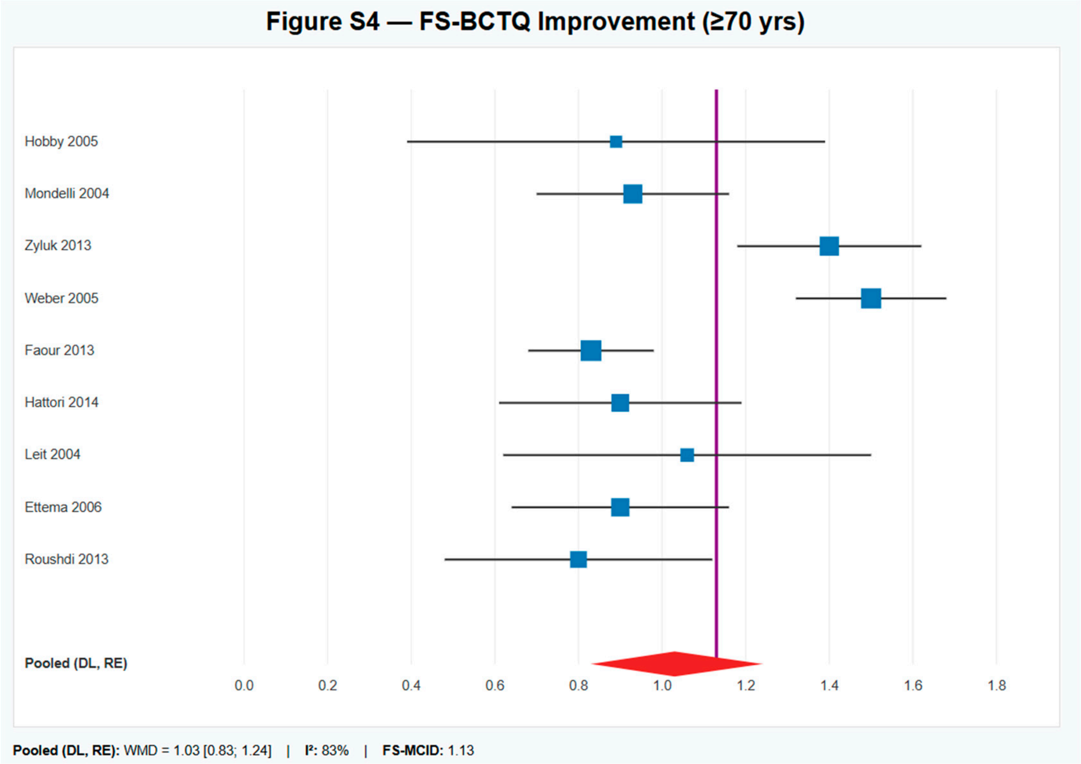

Figure S4 — FS-BCTQ Improvement, Elderly ≥ 70 yrs (9 Studies)

Study	n	WMD	95 % CI	Wt %
Hobby 2005 (>70)	14	0.89	[0.39; 1.39]	7.7
Mondelli 2004 G4	72	0.93	[0.70; 1.16]	12.1
Zyluk 2013 (>65)	77	1.40	[1.18; 1.62]	12.2
Weber 2005 (≥65)	92	1.50	[1.32; 1.68]	12.8
Faour 2013 (≥65)	52	0.83	[0.68; 0.98]	13.2
Hattori 2014 (octo.)	27	0.90	[0.61; 1.19]	11.3
Leit 2004 (≥70)	14	1.06	[0.62; 1.50]	8.6
Ettema 2006 (≥70)	47	0.90	[0.64; 1.16]	11.6
Roushdi 2013 (≥80)	24	0.80	[0.48; 1.12]	10.6
Pooled (DL, RE)	419	1.03	[0.83; 1.24]	—

Heterogeneity: $\tau^2 = 0.0754$; $Q = 46.33$ (df = 8); $I^2 = 83\%$

Overall effect: $Z = 9.97$ ($p < 0.0001$)

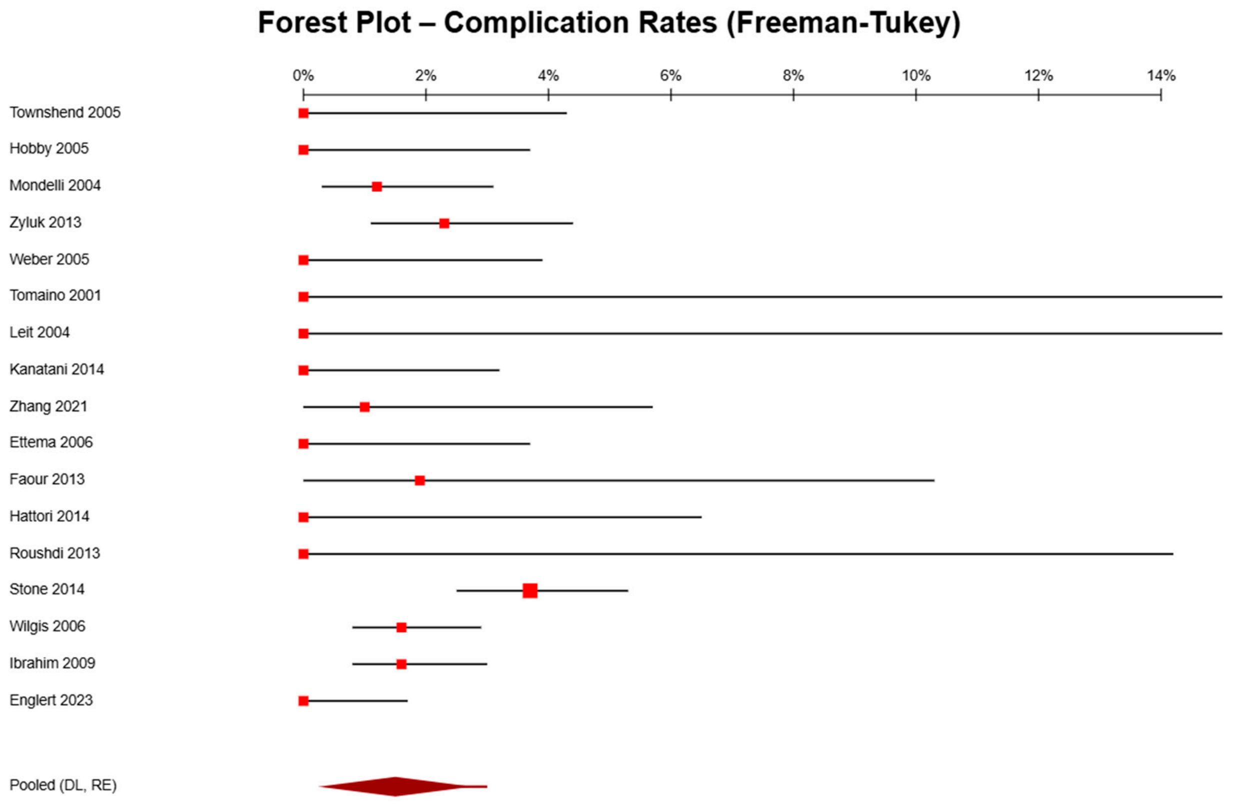

Figure S5 — Complication Rates (17 Studies, Freeman-Tukey)

Study	Ev	n	Rate	95 % CI
Townshend 2005	0	83	0.0%	[0.0; 4.3]
Hobby 2005	0	97	0.0%	[0.0; 3.7]
Mondelli 2004	4	323	1.2%	[0.3; 3.1]
Zyluk 2013	9	386	2.3%	[1.1; 4.4]
Weber 2005	0	92	0.0%	[0.0; 3.9]
Tomaino 2001	0	13	0.0%	[0.0; 24.7]
Leit 2004	0	14	0.0%	[0.0; 22.8]
Kanatani 2014	0	112	0.0%	[0.0; 3.2]
Zhang 2021	1	96	1.0%	[0.0; 5.7]
Ettema 2006	0	96	0.0%	[0.0; 3.7]
Faour 2013	1	52	1.9%	[0.0; 10.3]
Hattori 2014	0	55	0.0%	[0.0; 6.5]
Roushdi 2013	0	24	0.0%	[0.0; 14.2]
Stone 2014	28	756	3.7%	[2.5; 5.3]
Wilgis 2006	10	635	1.6%	[0.8; 2.9]
Ibrahim 2009	10	608	1.6%	[0.8; 3.0]
Englert 2023	0	214	0.0%	[0.0; 1.7]
Pooled (DL, RE)	63	3656	~1.5%	[~0.5; ~3.0]

Heterogeneity: Moderate-to-high I^2 driven mainly by Stone 2014 (3.7%) vs. multiple zero-event studies.

Cross-Figure Summary

Figure	Outcome	k	Pooled Estimate	95 % CI	I^2
S1	SS-BCTQ overall	11	WMD = 1.62 pts	[1.50; 1.74]	87%
S2	SS-BCTQ elderly	9	WMD = 1.61 pts	[1.43; 1.80]	78%
S3	FS-BCTQ overall	11	WMD = 1.03 pts	[0.89; 1.17]	91%
S4	FS-BCTQ elderly	9	WMD = 1.03 pts	[0.83; 1.24]	83%
S5	Complication rate	17	~1.5%	[~0.5; ~3.0]	Moderate

Key takeaways visible across all figures:

- SS improvement is consistently above the MCID of 1.05 in both overall and elderly subgroups → strong clinical signal.
- FS improvement (~1.03 pts) straddles the MCID of 1.13 → borderline, warrants cautious interpretation.
- Complication rate is low (~1.5%), dominated by zero-event studies; Stone 2014 is a visible outlier.
- All I^2 values are high (>75%), consistent with the GRADE LOW certainty rating noted in the code's footer annotation.

Author Contributions: Conceptualization, De Vitis R and Rocchi L; methodology, De Vitis R.; software, Cannella A; validation, Delia G and Cilli V; formal analysis, Cilli V; investigation, De Vitis R. and Sassara GM; resources, Cannella A; data curation, Di Dio E and Frittella G; writing original draft preparation, Di Dio E; writing review and editing, Sassara GM and Valerio M; visualization, Cannella A; supervision, Rocchi L; project administration, De Vitis R; funding acquisition, none. All authors have read and agreed to the published version of the manuscript.

Funding: This research received no external funding.

Institutional Review Board Statement: Not applicable.

Informed Consent Statement: Not applicable.

Data Availability Statement: none.

Acknowledgments: none.

Conflicts of Interest: The authors declare no conflicts of interest.

Abbreviations

The following abbreviations are used in this manuscript:
CTS Carpal Tunnel Syndrome
CTR Carpal Tunnel Release
PRISMA Preferred Reporting Items for Systematic Reviews and Meta-Analyses
BCTQ Boston Carpal Tunnel Questionnaire
MCID Minimally Clinically Important Difference
SS Symptom Severity
FS Functional Status
WMDs Weighted Mean Differences
CIs Confidence Intervals
GRADE Grading of Recommendations Assessment, Development and Evaluation
NOS Newcastle-Ottawa Scale
SWM Semmes-Weinstein monofilament
DASH Disability of the Arm, Shoulder and Hand
MHQ Michigan Hand Outcomes Questionnaire

REFERENCES

1. Bickel KD. Carpal tunnel syndrome. J Hand Surg Am. 2010;35(1):147–52.
2. Bland JD, Rudolfer SM. Clinical surveillance of carpal tunnel syndrome in two areas of the United Kingdom, 1991–2001. J Neurol Neurosurg Psychiatry. 2003;74(12):1674–9.
3. Osiak K, Elnazir P, Walocha JA, Pasternak A. Carpal tunnel syndrome: state-of-the-art review. Folia Morphol (Warsz). 2022;81(4):851–862.
4. United Nations, Department of Economic and Social Affairs, Population Division. World Population Prospects 2024: Summary of Results. UN DESA/POP/2024/TR/NO.9. New York: United Nations; 2024.
5. Pourmemari MH, Heliövaara M, Viikari-Juntura E, Shiri R. Carpal tunnel release: Lifetime prevalence, annual incidence, and risk factors. Muscle Nerve. 2018;58(4):497–502.
6. de Krom MC, Kester AD, Knipschild PG, Spaans F. Risk factors for carpal tunnel syndrome. Am J Epidemiol. 1990;132(6):1102–10.
7. Hobby JL, Venkatesh R, Motkur P. The effect of age and gender upon symptoms and surgical outcomes in carpal tunnel syndrome. J Hand Surg Br. 2005;30(6):599–604.
8. Georgiew F, Maciejczak A, Florek J. Results of surgical treatment of carpal tunnel syndrome. Ortop Traumatol Rehabil. 2014;16(5):455–68.
9. Graham B, Regehr G, Naglie G, Wright JG. Development and validation of diagnostic criteria for carpal tunnel syndrome. J Hand Surg Am. 2006;31(6):919–24.
10. Shapiro LM, Kamal RN; Management of Carpal Tunnel Syndrome Work Group; American Academy of Orthopaedic Surgeons. American Academy of Orthopaedic Surgeons/ASSH Clinical Practice Guideline Summary Management of Carpal Tunnel Syndrome. J Am Acad Orthop Surg. 2025;33(7):e356–e366.
11. Aghda AK, Asheghan M, Amanollahi A. Comparisons of electrophysiological and clinical findings between young and elderly patients with Carpal Tunnel Syndrome. Rev Neurol (Paris). 2020;176(5):387–392.
12. Blumenthal S, Herskovitz S, Verghese J. Carpal tunnel syndrome in older adults. Muscle Nerve. 2006;34(1):78–83.
13. Townshend DN, Taylor PK, Gwynne-Jones DP. The outcome of carpal tunnel decompression in elderly patients. J Hand Surg Am. 2005;30(3):500–5.
14. Seror P. Carpal tunnel syndrome in the elderly. "Beware of severe cases". Ann Chir Main Memb Super. 1991;10(3):217–25.
15. Kouyoumdjian JA. Carpal tunnel syndrome. Age, nerve conduction severity and duration of symptomatology. Arq Neuropsiquiatr. 1999;57(2B):382–6.
16. McQuarrie IG. Peripheral nerve surgery. Neurol Clin. 1985;3(2):453–66.
17. Verghese J, Galanopoulou AS, Herskovitz S. Autonomic dysfunction in idiopathic carpal tunnel syndrome. Muscle Nerve. 2000;23(8):1209–13.
18. D'Orio M, De Vitis R, Taccardo G, Rocchi L, Ferrari F, Perna A, Passiatore M. Clinical usefulness of nutraceutics with acetyl-L-carnitine, α-lipoic acid, phosphatidylserine, curcumin, C, E and B-group vitamins in patients awaiting for carpal tunnel release during COVID-19 pandemic: a randomized controlled open label prospective study. Acta Biomed. 2023;94(S2):e2023050.

19. Mondelli M, Padua L, Reale F. Carpal tunnel syndrome in elderly patients: results of surgical decompression. J Peripher Nerv Syst. 2004;9(3):168–76.
20. Zyluk A, Puchalski P. A comparison of the results of carpal tunnel release in patients in different age groups. Neurol Neurochir Pol. 2013;47(3):241–6.
21. Weber RA, Rude MJ. Clinical outcomes of carpal tunnel release in patients 65 and older. J Hand Surg Am. 2005;30(1):75–80.
22. Mondelli M, Padua L, Reale F, Signorini AM, Romano C. Outcome of surgical release among diabetics with carpal tunnel syndrome. Arch Phys Med Rehabil. 2004;85(1):7–13.
23. Thomsen NOB, Rosén I, Dahlin LB. Neurophysiologic recovery after carpal tunnel release in diabetic patients. Clin Neurophysiol. 2010;121(9):1569–1573.
24. De Vitis R, Passiatore M, Cilli V, Apicella M, Taccardo G. SARS-COV-2 infection and involvement of peripheral nervous system: a case series of carpal tunnel syndrome aggravation or new onset with COVID-19 disease and a review of literature. Georgian Med News. 2023;(340-341):61–66.
25. Rocchi L, De Vitis R, Pietramala S, Fulchignoni C, D'Orio M, Mazzone V, Marcuzzi A. Resurfacing Capitate Pyrocarbon Implant for the treatment of advanced wrist arthritis in the elderly: a retrospective study. Eur Rev Med Pharmacol Sci. 2022;26(1 Suppl):92–99.
26. Tomaino MM, Weiser RW. Carpal tunnel release for advanced disease in patients 70 years and older: does outcome from the patient's perspective justify surgery? J Hand Surg Br. 2001;26(5):481–3.
27. Leit ME, Weiser RW, Tomaino MM. Patient-reported outcome after carpal tunnel release for advanced disease: a prospective and longitudinal assessment in patients older than age 70. J Hand Surg Am. 2004;29(3):379–83.
28. Porter P, Venkateswaran B, Stephenson H, Wray CC. The influence of age on outcome after operation for the carpal tunnel syndrome. A prospective study. J Bone Joint Surg Br. 2002;84(5):688–91.
29. Englert CH, Hammert WC. Older Patients Demonstrate PROMIS Outcomes Comparable to Younger Cohorts After Carpal Tunnel Release. Hand (N Y). 2023;18(6):970–977.
30. Greenslade JR, Mehta RL, Belward P, Warwick DJ. Dash and Boston questionnaire assessment of carpal tunnel syndrome outcome: what is the responsiveness of an outcome questionnaire? J Hand Surg Br. 2004;29(2):159–64.
31. Witt JC, Hentz JG, Stevens JC. Carpal tunnel syndrome with normal nerve conduction studies. Muscle Nerve. 2004;29(4):515–22.
32. Bezerra P, Alves D. A relação entre autopercepção do estado de saúde e a condição física em septuagenários e octogenários. Cien Saude Colet. 2016;21(11):3525–3532.
33. Levine DW, Simmons BP, Koris MJ, Daltroy LH, Hohl GG, Fossel AH, Katz JN. A self-administered questionnaire for the assessment of severity of symptoms and functional status in carpal tunnel syndrome. J Bone Joint Surg Am. 1993;75(11):1585–92.
34. Naves TG, Kouyoumdjian JA. Carpal tunnel syndrome in the elderly: nerve conduction parameters. Arq Neuropsiquiatr. 2010;68(1):87–92.
35. Moola S, Munn Z, Tufanaru C, Aromataris E, Sears K, Sfetcu R, Currie M, Lisy K, Qureshi R, Mattis P, Mu P. Chapter 7: Systematic reviews of etiology and risk. In: Aromataris E, Lockwood C, Porritt K, Pilla B, Jordan Z, Munn Z, eds. JBI Manual for Evidence Synthesis. Adelaide: JBI; 2024.
36. De Kleermaeker FGCM, Boogaarts HD, Meulstee J, Verhagen WIM. Minimal clinically important difference for the Boston Carpal Tunnel Questionnaire: new insights and review of literature. J Hand Surg Eur Vol. 2019;44(3):283–289.
37. Mehta SP, Weinstock-Zlotnick G, Akland KL, Hanna MM, Workman KJ. Using Carpal Tunnel Questionnaire in clinical practice: a systematic review of its measurement properties. J Hand Ther. 2020;33(4):493–506.
38. Ozyurekoglu T, McCabe SJ, Goldsmith LJ, LaJoie AS. The minimal clinically important difference of the carpal tunnel syndrome symptom severity scale. J Hand Surg Am. 2006;31(5):733–738.
39. Kanatani T, Nagura I, Kurosaka M, Kokubu T, Sumi M. Electrophysiological assessment of carpal tunnel syndrome in elderly patients: one-year follow-up study. J Hand Surg Am. 2014;39(11):2188–91.
40. Zhang D, Earp BE, Benavent KA, Blazar P. Long-Term Outcomes and Mortality Following Carpal Tunnel Release in Patients Older Than 80 Years of Age. World Neurosurg. 2021;151:e1002–e1006.
41. Radwin RG, Sesto ME, Zachary SV. Functional tests to quantify recovery following carpal tunnel release. J Bone Joint Surg Am. 2004;86(12):2614–20.
42. Guyatt GH, Oxman AD, Vist GE, et al. GRADE: an emerging consensus on rating quality of evidence and strength of recommendations. BMJ. 2008;336(7650):924–926.

43. Ettema AM, Amadio PC, Cha SS, Harrington JR, Harris AM, Offord KP. Surgery versus conservative therapy in carpal tunnel syndrome in people aged 70 years and older. Plast Reconstr Surg. 2006;118(4):947–958.
44. Faour Martín O, Martín Ferrero MÁ, Valverde García JA, Sáez López MP, Vega Castrillo A, De La Red Gallego MÁ, Cortés Villar JM, Almaraz Gómez A. La neurólisis del nervio mediano en el paciente anciano: evaluación a largo plazo. Rev Esp Geriatr Gerontol. 2013;48(2):65–8.
45. Hattori Y, Doi K, Koide S, Sakamoto S. Endoscopic release for severe carpal tunnel syndrome in octogenarians. J Hand Surg Am. 2014;39(12):2448–53.
46. Roushdi I, Wakeling C, Clark DL. Patient-reported outcomes following carpal tunnel decompression in patients over 80 years old. J Hand Surg Eur Vol. 2013;38(5):565.
47. Stone OD, Clement ND, Duckworth AD, Jenkins PJ, Annan JD, McEachan JE. Carpal tunnel decompression in the super-elderly: functional outcome and patient satisfaction are equal to those of their younger counterparts. Bone Joint J. 2014;96-B(9):1234–8.
48. Wilgis EF, Burke FD, Dubin NH, Sinha S, Bradley MJ. A prospective assessment of carpal tunnel surgery with respect to age. J Hand Surg Br. 2006;31(4):401–6.
49. Ibrahim T, Majid I, Clarke M, Kershaw CJ. Outcome of carpal tunnel decompression: the influence of age, gender, and occupation. Int Orthop. 2009;33(5):1305–9.
50. Lander S, Lander A, Hammert WC. Outcomes of Patients with and without Nighttime Awakening Symptoms After Carpal Tunnel Release. Hand (N Y). 2022;17(1):23–27.
51. Sassara G, Cannella A, Caruso L, Taccardo G, Passiatore M, Militerno A, Marinangeli M, De Vitis R. Effectiveness of ActiPatch in the management and prevention of pillar pain following open carpal tunnel release surgery in weight-bearing hand of elderly patients: an observational study. Med Glas (Zenica). 2025;22(1):115–120.
